# The Impact of Surfactants in Tailoring Nanostructured Lipid Carriers for Inhaled Levofloxacin

**DOI:** 10.1021/acsomega.6c01967

**Published:** 2026-08-13

**Authors:** Viviane Lucia Beraldo de Araujo, Samira Elisa Alves Geraldo, Marcelo van Vliet Lima, Gabriel da Silva Cordeiro, João Paulo Guarnieri, Marcelo Lancellotti, Karina Cogo-Müller, Catarina Raposo, Laura Oliveira-Nascimento

**Affiliations:** † Faculdade de Engenharia Química, 28132 Universidade Estadual de Campinas, Campinas 13083-852, Brazil; ‡ Faculdade de Ciências Farmacêuticas, 28132 Universidade Estadual de Campinas, Campinas 13083-871, Brazil; § Faculdade de Ciências Médicas, Universidade Estadual de Campinas, Campinas 13083-888, Brazil; ∥ Sanofi Medley Farmacêutica Ltda, Campinas 13080-010, Brazil

## Abstract

Nanoparticles can enhance pulmonary delivery of antibiotics by modifying or targeting drug release, increasing drug stability, decreasing mucus entrapment, disrupting biofilms, and reaching the deep lung. Surfactants play a key role as coating agents for several nanoparticle types, modulating interfacial interactions with drugs and the environment. Specifically, levofloxacin (LV) inhalation can benefit from its loading into nanostructured lipid carriers (NLCs) for high drug entrapment, sustained release, and antimicrobial efficacy, but no studies have focused on the relevance of the surfactant type on its biological and physicochemical performance. Therefore, this study aimed to evaluate the impact of three nonionic surfactants—polysorbate 80 (P80), poloxamer 407 (P407), and poloxamer 188 (P188)—on the physicochemical and biological *in vitro* performance of LV-loaded NLCs. NLCs presented similar physicochemical properties, with average sizes of 100–200 nm, polydispersity <0.3, slightly negative ζ-potential (−5 to −19 mV), high drug entrapment efficiency (>77%), and rounded-shape morphology. The P188 coating tends to have a prolonged release profile compared to the other formulations, but further studies should be conducted to confirm this tendency. All formulations preserved the antimicrobial activity of LV *in vitro* compared to the free LV against *Klebsiella pneumoniae*, *Staphylococcus aureus*, *Burkholderia cepacia*, and *Haemophilus influenzae*. Notably, P407 was the only surfactant able to maintain the biofilm inhibition of *B. cepacia* promoted by LV up to half the minimum inhibitory concentration (MIC) under the tested conditions. This is the first evidence of LV efficacy against biofilms of this species, highlighting the importance of LV application against this undervalued but deadly infection agent in patients with cystic fibrosis. Concerning device compatibility, P407-stabilized NLCs retained their size and PDI within the same range after nebulization through a vibrating mesh, which is favorable for future pulmonary delivery. In summary, these findings highlight the importance of surfactant type selection, indicating the preliminary feasibility of the P407-based formulation and providing a foundation for future investigations on the pulmonary delivery of LV.

## Graphical abstract

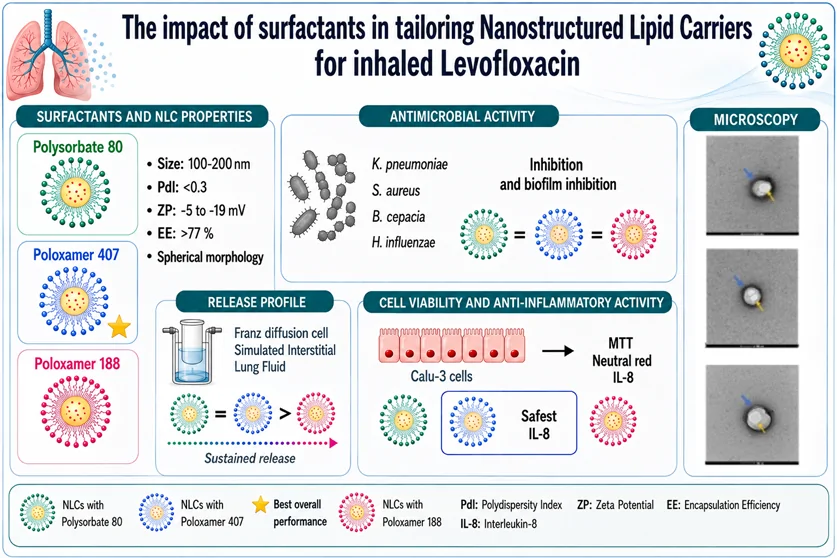

## 1 Introduction

Respiratory tract infections remain a significant cause of disease and death worldwide. Among bacterial etiologies, *Streptococcus pneumoniae*, *Staphylococcus aureus*, and *Klebsiella pneumoniae* predominate in microbiological diagnoses.^1^ Markedly, *Pseudomonas aeruginosa* is the primary pathogen in lung infections among cystic fibrosis patients, increasing the mortality of this population.^2^ Nevertheless, pathogens like the *Burkholderia cepacia* complex have increased in frequency and pulmonary infection relevance, with fewer standardized antibiotics available for their treatment.^3^

Levofloxacin (LV), a broad-spectrum antibiotic, eliminates most of the bacterial species that cause community-acquired pneumonia and acute exacerbations of chronic bronchitis.^4^ In addition, it is highly effective against *P. aeruginosa* and classified as an essential drug to treat multidrug-resistant tuberculosis.^5^ This outstanding performance is overshadowed by increased antimicrobial resistance (still low compared to other antibiotics),^6^ besides relevant adverse events (grades 3–5) due to systemic distribution.^5^

Lung delivery can overcome these limitations by enhancing local delivery and accelerating the onset of action, with the added benefit of lower drug metabolism.^7^ This rationale is supported by the FDA approval of inhaled formulations of Tobramycin, Amikacin sulfate, and Aztreonam.^8^ Inhaled LV has also been approved by the European Union and Canadian health agencies to treat *Pseudomonas* infections in cystic fibrosis patients. Pulmonary delivery of LV allows dosage fractionation with efficient bacterial clearance and fewer side effects. However, it causes dysgeusia (taste disturbance) and cough, which were not observed with oral administration.^9^ Specifically, commercial pulmonary LV utilizes magnesium chloride to prolong pulmonary retention, but the bitter taste probably contributes to dysgeusia.^10^

Nanoparticles (NPs) can increase the lung retention of drugs, generally for longer durations than complexation with salts, but the formulation process is not straightforward. For instance, LV-loaded anionic liposomes exhibited prolonged release over 72 h, maintaining antibacterial activity against *P. aeruginosa*, whereas PLA-*g*-PEG NPs failed to modify drug release and increased the minimum inhibitory concentration (MIC) value.^2^ Currently, inhaled liposomal amikacin is considered more efficient than the free drug in reducing *Pseudomonas* burden (preclinical), presenting sustained activity for up to 24 months in a clinical trial.^11^

The inhalation market is strongly focused on liposome delivery, with commercial products and ongoing clinical trials, but nanostructured lipid carriers (NLCs) can provide better drug and colloidal stability than liposomes.^12^ NLCs coated with P188 and carrying LV with DNase have shown a sustained release profile, effective antimicrobial activity against *P. aeruginosa* and *S. aureus*, as well as inhibition of bacterial biofilms, even with an average encapsulation efficiency of 56%.^13^ Our previous work optimized LV in NLCs with P80, testing different lipids in pre-formulation, and different process parameters, reaching an optimized encapsulation efficiency (70–85%). However, we did not vary the surfactant type nor study long term stability.^14,15^ To the best of our knowledge, NPs loaded with LV have not reached the market or clinical trials yet, regardless of the type.

Some of the most common surfactants in NP formulations are poloxamer 188, 407, and polysorbate 80 (P188, P407, and P80, respectively).^7,16^ These three surfactants are nonionic and generally less toxic than ionic surfactants,^17,18^ providing hydrophilic coatings that can avoid nanoparticle trapping in the mucus layer.^19^ Indeed, the P407 coating of PLGA nanoparticles increased the mucus penetration rate and velocity *ex vivo*, which contributed to a higher lung retention time of dexamethasone in mice. Although P188 did not enhance the same parameters in this study, its concentration was not sufficient for surface shielding, as evidenced by the negative zeta potential.^20^ P80 was also able to decrease interactions with mucin in vitro, though it was inferior to P407 for shielding curcumin nanocrystals.^19^

Considering the lack of information on surfactant coating and antibacterial or lung cell properties of LV-loaded NLCs, this study evaluates the impact of three nonionic surfactant types (P80, P407, and P188) on NLC performance. After comparing physicochemical parameters, we assessed antimicrobial activity against bacteria that infect the lungs and evaluated *in vitro* cell viability (Calu-3 lung cells), utilizing IL-8 modulation as an inflammation marker.

**Table 1:** List of Reagents and Specifications^a^

Category	Reagent	Catalogue #	Company/Country
Drug	Levofloxacin hemihydrate (LV)	050,100,078	Sanofi-Medley Farmacêutica Ltd.a, Brazil
Excipient	Super Refined polysorbate-80	SR48833	Croda, Brazil
	Super Refined oleic acid	SR40211	Croda, Brazil
	Precirol ATO 5	3092	Gattefossé, France
	Kolliphor P 188 Geismar (Poloxamer 188, P188)	292,618	BASF, Brazil
	Pluracare F 127 NF (Poloxamer 407, P407)	30,164,505	Chemspecs, Brazil
Microbiological assays	Trypticasein soy agar (TSA)	K25-1068	KASVI, Brazil
	Mueller Hinton Broth (MHB)	275,730	BD Difco, Brazil
	Brain Heart Infusion Broth (BHI)	K25-1400	Kasvi, Brazil
	Resazurin	R07505RA	Êxodo Científica, Brazil
Biological assays	Lipopolysaccharides (LPS) from *Escherichia coli*	00-4976-93	Invitrogen, USA
	Human IL-8/CXCL8 DuoSet ELISA	DY208	R&D Systems, USA
	Dulbecco’s Modified Eagle Medium (DMEM)	D6429	Sigma-Aldrich, USA
	Modified Eagle Medium (MEM) nonessential amino acids	M7145	Sigma-Aldrich, USA
	Fetal bovine serum (FBS)	63	Cultilab, Brazil
	Gibco Trypsin-EDTA (0.25%)	25,200,056	ThermoFisher Scientific, Germany
	Hank’s balanced salt solution	H8264	Sigma-Aldrich, USA
	3-(4,5-Dimethylthiazol-2-yl)-2,5-diphenyltetrazolium bromide (MTT)	298-93-1	Sigma-Aldrich, USA
	Neutral red	QHV130-25g	QHEMIS,Brazil

a All other chemicals and solvents were of analytical grade.

## 2 Material and Methods

### 2.1 Material

Reagents and kits were selected as described in Table 1.

#### 2.1.1 Bacterial Strains

*Klebsiella pneumoniae* (ATCC BAA 1705 and ATCC 700603), *Staphylococcus aureus* (ATCC 29213 and ATCC 33591), *Burkholderia cepacia* (ATCC 25416), and *Haemophilus influenzae* (ATCC 51907) were used to conduct microbiological assessments.

#### 2.1.2 Cell Line

Calu-3 is an epithelial cell line derived from a human bronchial adenocarcinoma. It was purchased from Banco de Células do Rio de Janeiro – BCRJ (Brazil) (BCRJ code 0264, reference ATCC HTB-55). Passages used: 40–49. Clinical trial number: not applicable.

### 2.2 NLC Production

NLCs were prepared via the hot emulsification-ultrasonication method, as described previously.^15^ The lipid phase (70% w/w of Precirol ATO 5 and 30% w/w of oleic acid, corresponding to 10% w/w of the final formulation) was melted in a water bath at 58 ± 1 °C under magnetic stirring (300 rpm), followed by the addition of 0.5% w/w of LV. The aqueous phase (P80, P407, or P188 at 4% w/w in ultrapure water), previously heated under the same conditions, was added to the lipid phase under mixing in an Ultraturrax blender (IKA T18 basic, Germany) at 12,000 rpm for 3 min using the S18N-19G dispersing tool. This emulsion was sonicated for 20 min in cycles of 30 s (on/off) at an amplitude of 50% to achieve nanometric and homogeneous particle size, using a tip sonicator (Vibracell, Sonics & Materials Inc., USA) fitted with a 3 mm probe, with a power of 130 W and a nominal frequency of 20 kHz. The final dispersion was cooled over an ice bath to 25 °C and stored at room temperature (RT), protected from light.

### 2.3 NLC Physicochemical Characterization

#### 2.3.1 Determination of Hydrodynamic Diameter (Z-Average), Polydispersity Index (PDI), Zeta Potential (ZP), and Nanoparticle Concentration

NLCs were analyzed by Dynamic Light Scattering (DLS) (Zetasizer Nano ZS90, Malvern Instruments Ltd, UK) at a 90 ° scattering angle and 25 °C, using a disposable polystyrene cuvette for determining measurements of z-average and PDI based on the intensity of light scattering. Samples were diluted to 1:200 in 10 mM sodium chloride to achieve an adequate correlation coefficient (between 0.7 and 1). The zeta potential (ZP) was determined by measuring the electrophoretic mobility using a disposable polystyrene cuvette model DTS1070 with electrodes. All samples were measured in triplicate, and results were presented as mean ± standard deviation. DLS allowed the obtention of size distribution of D10/D50/D90 based on volume.

For nanoparticle tracking analysis, (NTA) using the Nanosight instrument (Malvern Instruments Ltd.), samples were diluted in ultrapure water (dilution factor: 15,000x) and injected. The images were captured during 30 s from five different populations of each NLC at RT. NTA allowed the determination of nanoparticle concentration (nanoparticles per mL) and size values of D10/D50/D90.

#### 2.3.2 Entrapment Efficiency (EE)

The LV was quantified by high-performance liquid chromatography (HPLC) as described in the United States Pharmacopoeia (USP) monograph for Levofloxacin Tablets,^21^ using a Shimadzu HPLC system (Prominence-i LC2030C, Shimadzu, Japan). The mobile phase consisted of a buffer (8.5 g/L of ammonium acetate, 1.25 g/L of cupric sulfate pentahydrate, and 1.3 g/L of l-isoleucine in water) and methanol in a ratio of 7:3. The column contained the L1 packing (Waters Symmetry C18 250 mm × 4.6 mm i.d. column, 5 μm particle size). The analysis occurred at 45 °C, with a flow rate of 0.8 mL/min, an injected sample volume of 25 μL, detection at 360 nm, and a running time of 26 min. The calibration curve using LV standard ranged from 5 to 200 μg//mL (*r* ^2^ = 0.9999). The limits of detection and quantification were found to be 1.97 μg/mL and 5.97 μg/mL, respectively.^15^

The entrapment efficiency (EE) was indirectly determined using the ultrafiltration method: 500 μL of NLC suspension was centrifuged at 4100*g* for 20 min in an Eppendorf 5418 centrifuge (Germany), using centrifugal filter tubes with a 30 kDa MW cutoff (Millex, Millipore, USA).^15^ The supernatant was diluted 25 times in the mobile phase and quantified by HPLC. EE was calculated using eq 1. LV in the samples was quantified in triplicate, and the results were presented as mean ± standard deviation.

1 $$EE\left(\%\right)=\frac{Total\;amount\;of\;drug-free\;drug}{Total\;amount\;of\;drug}\times 100$$

#### 2.3.3 Stability Test

The NLCs were stored for 90 days in a stability test chamber at 40 °C and 75% RH, protected from light in type I glass vials closed with rubber stoppers and wrapped in aluminum foil. Hydrodynamic diameter (z-average), polydispersity index (PDI), zeta potential (ZP), and entrapment efficiency (EE) were determined as described previously, both on the day of preparation (fresh sample) and after 90 days of storage. The results were presented as mean ± standard deviation.

#### 2.3.4 Morphology and Integrity Characterization of NLCs by Electron Microscopy

NLCs were submitted to TEM analysis (100 times dilution in ultrapure water) by depositing 10 μL onto copper grids coated with carbon film (200 mesh). Excess sample was removed using filter paper, and water was evaporated for 1 h at RT. Grids were treated with 20 μL of 2% uranyl acetate for 1 min to provide contrast, followed by excess removal. Then, grids were washed with 20 μL of ultrapure water for 1 min and dried with filter paper at RT for 24 h before analysis. Micrographs were obtained using a Tecnai G2 Spirit BioTWIN transmission electron microscope, operated at 80 kV and magnifications of 30,000× and 68,000× (adapted methodology).^22^

Cryo-TEM was applied to obtain information on the morphology, size, and integrity information on the native structure of the NLC (without fixation, dehydration, or staining/contrast agents) by a fast-freezing sample step. Copper grids with carbon film type Lacey, 300 mesh (#01895-F, Ted Pella, EUA), were treated with a charge of 25 mA per 50 s, in the EasiGlow (I) (Ted Pella, EUA). The grids were placed in the sample vitrification robot Vitrobot Mark IV (Thermo, EUA). Nondiluted samples were applied onto the grid, and excess was removed by blot time 4 and blot force −3. The grids were immediately frozen in liquid ethane and kept in liquid nitrogen until analysis. Images were acquired using a TEM model Talos Arctica (Thermo, EUA), operated at 200 kV. The microscope is equipped with a Ceta 16 M 4k x 4k camera (Thermo, EUA) for the acquisition of digital images.

### 2.4 Drug Release Profiles

Drug release was performed using static vertical diffusion Franz cells with automatic sampling (Microette Plus, Hanson Research, USA) under sink conditions (more than 3 times the LV solubility). The receptor chamber held 7 mL of Simulated Interstitial Lung Fluid (SILF, composition as described in the reference)^2^ at 37 °C, stirred at 700 rpm, while the donor chamber contained 1 mL of sample diluted in SILF. The diffusion surface area was 1.76 cm^2^, covered by a regenerated cellulose membrane of 12–14 kDa (Servapor dialysis tubing). Aliquots of 2.5 mL were collected at specific time intervals and replaced with SILF. The samples were analyzed by using HPLC. The similarity factor (F2) was determined according to U.S. Food and Drug Administration (FDA) guidelines to compare dissolution profiles, by the eq 2: *n* is the number of sampling time points, *R*_*t*_ is the percentage of drug dissolved from the reference formulation at time (*t*), and *T*_*t*_ is the percentage dissolved from the test formulation at *t*. Values of 50 < *f*2 < 100 indicate profile similarity.

2 $$f2=50.\log\left\{{[1+\frac{1}{n}\sum_{t=1}^{n}{\left(R_{t}-T_{t}\right)}^{2}]}^{0.5}\times 100\right\}$$

### 2.5 Determination of Minimum Inhibitory Concentration (MIC)

Bacteria were cultured (37 °C/24 h) on tryptic soy agar (TSA) before experiments. Isolated colonies were suspended in NaCl 0.9%, and the inoculum was adjusted following the broth microdilution protocol from the Clinical and Laboratory Standards Institute (CLSI)^23^ for MIC determination. Tests were conducted using Mueller Hinton Broth (MHB) as the culture medium and supplemented with Hemin and NAD at 10 μg/mL for *Haemophilus influenzae*. Stock solutions of LV and NLCs were prepared in MHB to obtain the same corresponding LV concentration. The treatments prepared were LV aqueous solution, NPLBL, NPLLV, P80, P407, and P188 diluted in cell culture medium within a 96-well microplate, followed by the addition of 5 × 10^4^ CFU/well of bacteria. Sterility control (MHB) and growth control were performed for each test. After incubation (37 °C/24h), bacteria were treated with 30 μL of 0.01% resazurin solution for 2 h to allow visualization of bacterial growth or its inhibition (pink and blue, respectively), enabling MIC determination. MIC assays were run in triplicate, in at least two independent experiments.

### 2.6 Inhibition Biofilm Assay

First, a suspension was prepared with *Burkholderia cepacia* ATCC25416 (5 × 10^7^ CFU/mL in Brain Heart Infusion broth (BHI) with 1% Glucose w/v). NPLLV formulations were diluted in culture medium from 8x MIC to 1/8x MIC range (MIC = 0.5 μg/mL). Then, 200 μL of the suspension was added to each well of a 96-well round-bottom plate and incubated at 37 °C for 24 h. The cultivated biofilms were stained with Crystal Violet (CV). For this, wells were washed three times with 200 μL of distilled water, dried for 30 min, and stained with 150 μL of CV (0.3% w/v in ultrapure water) for 30 min. The wells were washed again as previously described, and 200 μL of 98% (v/v) ethanol solubilized the bound CV. The content was transferred to a 96-well flat-bottom plate, and absorbance was measured at a wavelength of 575 nm using a microplate reader (Bio-Tek Instruments, Inc., Epoch, USA). Growth inhibition was described as a percentage of the positive control growth.

### 2.7 Calu-3 Cell Assays

#### 2.7.1 Calu-3 Undifferentiated Assays

Calu-3 was cultivated in DMEM supplemented with 10% (V/V) FBS (Cultilab, Brazil) and 1% (V/V) nonessential amino acids solution. Calu-3 was cultivated in cell culture flasks of 75 cm^2^ surface area, incubated at 37 °C, 5% CO_2_ and 90% relative humidity. The medium was changed every 2–3 days and subcultured after reaching ∼80% confluency, using a Trypsin-EDTA (0.25%) solution. Passages used in the experiments: 40–49 (maximum eighth passage post-thaw).

For cell viability assays, Calu-3 cells were seeded in a 96-well microplate at a density of 5 × 10^4^ cells/well (100 μL) and incubated for 24 h. Then, the cells were exposed to different treatments at different concentrations diluted in cell culture medium, including a viable cell control (only DMEM) and a dead cell control (DMSO 30% in DMEM). Cell viability was evaluated after 24 or 48 h by two different approaches: cell mitochondrial activity via 3-(4,5-dimethylthiazol-2-yl)-2,5-diphenyltetrazolium bromide (MTT) assay and cell membrane integrity via the neutral red assay (*n* = 4 replicates, minimum of two independent experiments).

For the MTT assay, the medium was removed from the wells and replaced with 110 μL of MTT (0.5 mg/mL in DMEM), followed by incubation for 3 h, removal, and treatment with 100 μL of isopropanol to dissolve the formazan crystals. The absorbance was read using a Multiskan GO microplate spectrophotometer at 570 nm wavelength (Thermo Fisher Scientific, Inc., Waltham, MA, USA).^23^

The Neutral Red assay was adapted^23,24^ and performed by removing the medium from the treated cells, washing with phosphate-buffered saline (PBS) (150 μL/well), adding 200 μL/well of neutral red solution (50 μg/mL, diluted in DMEM), and incubating for 3 h. Cells were washed with PBS again (150 μL/well) and treated with 150 μL of acidic-ethanol solution (1% glacial acetic acid, 50% of 96% ethanol, and 49% ultrapure water) to solubilize the cell-incorporated dye. The microplate was stirred for 10 min at 150 rpm and 37 °C in a shaker incubator. Absorbance was read at 540 nm. Results are expressed as mean ± standard deviation, obtained from at least 2 independent assays, with *n* ≥ 3 replicates per assay.

#### 2.7.2 Calu-3 Differentiation and Viability Assays

Calu-3 differentiation was assessed through cell viability (neutral red assay) and IL-8 cytokine secretion, and further characterized by scanning electron microscopy and Hoechst nuclear staining (Supporting Figure 4). Calu-3 differentiation followed the literature^23,25–27^ and was performed in Transwell-Clear inserts (6.5 mm, 0.4 μM diameter poresize, polyester membrane, 0.33 cm^2^ effective area for growth) over a 24-well microplate (Corning Costar, Cambridge, MA). Before cell seeding, inserts were washed with PBS, and 600 μL of cell culture medium were added to the basolateral compartment. Then, 100 μL of Calu-3 cells were seeded into the apical compartment (5 × 10^5^ cells/cm^2^, approximately 1.5 × 10^5^ cells/insert).

Cells were incubated, and the culture medium was changed every other day until cell monolayer transepithelial electrical resistance (TEER, measured using the Merck Millicell- ERS2 Electrical Resistance System Volt-Ohm meter, Germany) reached ≥300 Ω cm^2^, as indicative of a tight monolayer.^28^ Then, the medium was aspirated from the apical compartment and kept only in the basolateral compartment. The apical compartment was washed with 100 μL/insert of PBS every 2–3 days to remove excess mucus. Cells were incubated under air–liquid interface (ALI) for 12–16 days to allow differentiation.

TEER was measured after replacing the culture medium with PBS (500 μL basolateral +200 μL apical), incubating the samples for 30 min at 37 °C to stabilize the cell monolayer, inserting electrodes, and reading the values. TEER values were corrected by subtracting the mean resistance of the PBS-blank porous membranes^29^ (eq 3). TEER was also measured before and after treatments. Cell differentiation was also checked by scanning electron microscopy with Hoechst-33342 nuclei stain (Supporting Figure 4).

3 $$TEER\left(ohm\;x\;cm^{2}\right)=\left\{TEER\;cells\left(ohm\right)-TEER\;blank\left(ohm\right)\right\}\times 0.33\left(cm^{2}\right)$$

For cell viability, a neutral red assay was performed to verify whether the results were consistent with those from nondifferentiated Calu-3 cells (stimulated with LV aqueous solution, NPLLV_034, NPLBL_034). Cell viability control was evaluated with DMEM. 100 μL of each treatment was plated onto the apical compartment and incubated for 48 h. The following steps were similar to those for the nondifferentiated cells, except for shaking at 200 rpm to ensure better homogenization of the neutral red. 100 μL of the final content was transferred to a 96-well microplate to measure absorbance at 540 nm.^23,24^

#### 2.7.3 IL-8 Cytokine Quantification by Enzyme-Linked Immunosorbent Assay (ELISA)

Interleukin 8 (IL-8) produced by differentiated cells (apical and basolateral compartments) was quantified by immunoassay kits following the manufacturer’s instructions (Human IL-8/CXCL8 R&D Systems DuoSet ELISA). In brief, a monoclonal antibody against IL-8 was previously incubated overnight in the 96-well microplate. Standard curves, controls, and samples (concentrated or diluted) were added to the wells in duplicates. After washing the plate, a substrate solution was added. The values were read by the standard curve in a Multiskan GO microplate spectrophotometer at a wavelength of 540 nm (Thermo Fisher Scientific, Inc., Waltham, MA, USA) in duplicates and two independent experiments. The Calu-3 secretion of IL-8 was compared among control cells, cells treated with formulations (free LV solution, NPLLV, NPLBL, P80, P407, and P188), and stimulated cells with lipopolysaccharide (LPS) (with and without the treatments). Results are presented as mean ± standard deviation.

### 2.8 Statistical Analysis

Results were expressed as mean ± standard deviation (DLS, NTA, EE, stability tests, cell viability, and IL-8 quantification). The number of replicates and independent experiments was specified in each corresponding section. Statistical analyses were selected according to the experimental design. Cell viability data were analyzed by the nonparametric Mann–Whitney test. Symbols *, **, *** and **** represent statistical significance between the groups tested and the control (*p* < 0.05, *p* < 0.005, *p* < 0.0005, and *p* < 0.00005, respectively). The analysis involving two groups was performed with an unpaired *t*-test. Biofilm data were analyzed using two-way analysis of variance (ANOVA), followed by Tukey’s post hoc test for multiple comparisons. All analyses were performed using GraphPad Prism 8.0.1 software (GraphPad Software, San Diego, CA, USA). The differences were considered significant at *p* < 0.05.

## 3 Results and Discussion

### 3.1 NLC Production and Characterization

Surfactants play a key role in NLC formulations as surface-active agents or emulsifiers. Since they are amphiphilic, containing a hydrophilic (polar) “head” and a hydrophobic (apolar) “tail”, they can organize and stabilize lipid nanoparticles in a colloidal system. Nonionic emulsifiers can provide steric stabilization to the nanoparticles, avoiding their aggregation,^30^ which is different from charged-stabilization provided by ionic ones. NLCs were produced and evaluated with three nonionic surfactants: polysorbate 80 (P80), poloxamer 407 (P407), and poloxamer 188 (P188). All of them are considered oil-in-water (o/w) emulsifiers, since they present hydrophilic–lipophilic balance (HLB) values higher than 8 (15, 22, and 29, respectively),^31^ which is appropriate for NLC formulations. NLCs were characterized by different techniques to measure their particle size, size distribution, particle concentration number, and encapsulation efficiency (Tables 2 and 3).

**Table 2:** Physicochemical Characterization of Nanoparticles by DLS and HPLC^a^

Surfactant	Formulation Tracking Code	Size (nm)	PDI	Zeta Potential (mV)	**D10 (nm)**	**D50 (nm)**	**D90 (nm)**	**EE (%)**
P80	NPLBL_033	181 ± 8	0.246±0.011	-10 ± 1	97 ± 4	199 ± 11	438± 30	-
P80	NPLLV_033	137 ± 4	0.238 ± 0.031	-19 ± 3	75 ± 5	158 ± 9	314 ± 17	77 ± 3
P407	NPLBL_034	139 ± 5	0.152 ± 0.016	-5 ± 2	87 ± 3	151 ± 9	261 ± 20	-
P407	NPLLV_034	113 ± 1	0.138 ± 0.018	-9 ± 1	71 ± 1	121 ± 2	210± 7	84 ± 1
P188	NPLBL_035	158 ± 11	0.226 ± 0.018	-7 ± 1	87 ± 14	182 ± 14	371 ± 43	-
P188	NPLLV_035	111 ± 16	0.211 ± 0.040	-13 ± 1	63 ± 14	96 ± 18	259 ± 25	85 ± 1

a NPLBL = blank nanoparticles. NPLLV= levofloxacin-loaded nanoparticles. The number in tracking codes relates to the type of formulation (NP). Size (Z-average) = intensity-based hydrodynamic diameter; PDI = polydispersity index of the diameter. D10, D50, and D90 correspond to the volume-based particle sizes below which 10%, 50%, or 90% of the nanoparticles are smaller, respectively (*n* = 3, three separately prepared batches). EE = entrapment efficiency (*n* = 3, three separately prepared batches). All analyses are expressed as mean ± standard deviation. Graphs are available in Supporting Figure 1.

**Table 3:** Physical Characterization of Nanoparticles by NTA^a^

Surfactant	Formulation Tracking Code	Size (nm)	D10 (nm)	D50 (nm)	D90 (nm)	Span	Concentration (no Particles/mL)
P80	NPLBL_033	122 ± 4	78 ± 3	109 ± 6	188 ± 5	1.01	2.87 × 10^13^ ± 8.18 × 10^11^
P80	NPLLV_033	126 ± 8	77 ± 6	113 ± 7	189 ± 17	0.99	1.37 × 10^13^ ± 1.08 × 10^13^
P407	NPLBL_034	100 ± 1	67 ± 2	87 ± 2	153 ± 4	0.99	2.32 × 10^13^ ± 6.30 × 10^11^
P407	NPLLV_034	114 ± 6	73 ± 7	105 ± 9	169 ± 11	0.91	4.65 × 10^12^ ± 2.41 × 10^12^
P188	NPLBL_035	135 ± 4	92 ± 4	129 ± 2	183 ± 9	0.71	1.68 × 10^13^ ± 5.40 × 10^11^
P188	NPLLV_035	134 ± 1	98 ± 1	122 ± 1	188 ± 7	0.74	2.02 × 10^13^ ± 4.46 × 10^11^

a NPLBL = blank nanoparticles. NPLLV= levofloxacin-loaded nanoparticles. The number in tracking codes relates to type of formulation (Mean = hydrodynamic diameter; D10, D50, and D90 correspond to the particle size below which 10%, 50%, or 90% of the nanoparticles are smaller, respectively (*n* = 5). All analyses are expressed as mean ± standard deviation. Span = (D90 – D10)/D50. Graphs are available in Supporting Figure 2.

The NLCs showed high and similar EE values (77–85%), an acceptable PDI range (<0.3), and an average size suitable for the pulmonary route (≤300 nm, while remaining on average above 100 nm to favor local retention in the lungs).^32,33^ The NLC coated with P80 was consistent with our previous work, which provided a complete physicochemical characterization.^14^

DLS indicated a general decrease in mean particle size with LV incorporation, a trend not observed with NTA. This difference arises from the different measurement principles of the techniques. In DLS, particles are illuminated by a laser, and the hydrodynamic diameter is correlated with the intensity of scattered light. This principle suits monodisperse samples but emphasizes larger particles in polydisperse ones. In contrast, NTA calculates the size of individual particles from their Brownian movement, using the Stokes–Einstein equation similarly to DLS, but making it less influenced by the presence of larger particles within the population.

Both techniques revealed a broader particle size distribution for P80-based NLCs compared to those with P188 and P407, as indicated by higher PDI and span values. This finding may be attributed to the lower molecular weight of P80 relative to that of the poloxamers. Although the surfactant mass fraction was kept constant across formulations, the molar concentration of P80 was up to ten times higher than that of P188 and P407. This excess of P80 molecules may lead to surface saturation, potentially promoting interfacial rearrangements that result in increased particle size and heterogeneity.

DLS graphs (Supporting Figure 1) revealed a minor population of micrometer-sized particles in the P80 formulation, contributing to higher D90 and PDI values.

However, this population was not present in the NTA measurements. In general, D10/D50/D90 values from DLS were broader than those from NTA (Table 3), likely because of the intensity-weighted nature of DLS, which amplifies the contribution of larger particles rather than reflecting individual nanoparticle sizes.

The zeta potential is related to the colloidal stability of nanoparticles; however, in systems stabilized by nonionic surfactants, stability is mainly governed by steric hindrance rather than electrostatic repulsion. Therefore, such systems can remain stable even with a ζ-potential value below |20 mV|.^34^ There are higher ζ-potential values, in modulus, in NPLLVs compared to their respective placebos, suggesting a contribution of LV to the nanoparticle surface charge, possibly through surface adsorption (Table 2). Overall, the values ranged from slightly negative to nearly neutral, which favors better distribution of the drug in the lungs.^32^

### 3.2 Nanoparticle Morphology

Morphology was assessed by transmission electron microscopy (TEM) and cryogenic transmission electron microscopy (cryo-TEM). Cryo-TEM analysis of blank and LV-loaded nanoparticles with P80 (Figure 1a-d) showed mainly round-shaped nanoparticles with sizes similar to those obtained from DLS analysis. The structure showing two lamellae of the NLCs (Figure 1a-d) corroborates with our previous results using sorption and desorption analysis and X-ray diffraction (nanoparticles with P80).^14^ NLCs presented smaller sizes in TEM compared with cryo-TEM due to shrinkage upon drying during sample preparation.

**Figure 1:**
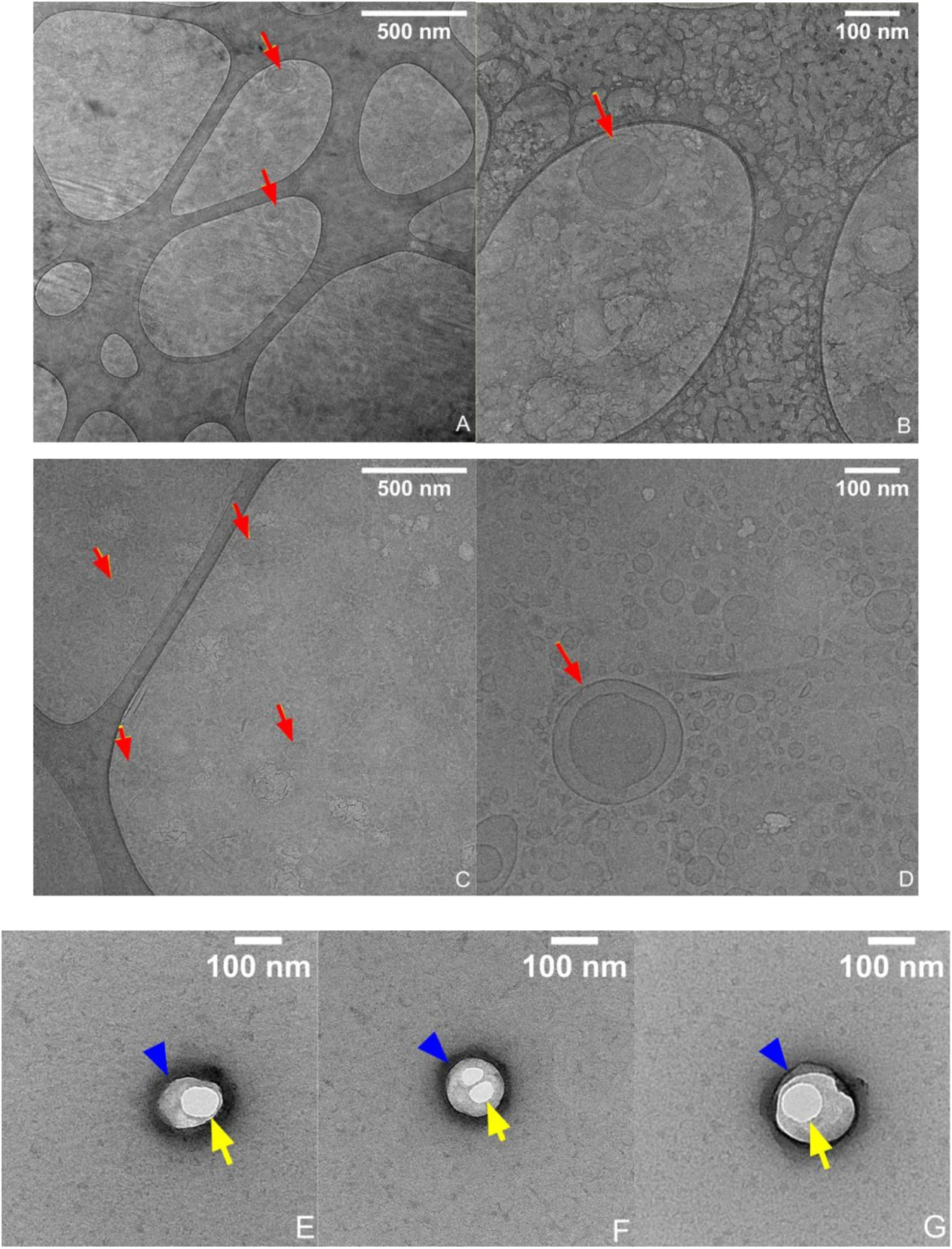
Transmission electron microscopy (TEM) micrographs of NLCs. Cryogenic-TEM images of nondiluted and nonstained (a, b) NPLBL (blank NLC) and (c, d) NPLLV_033 (NLC with levofloxacin), both produced with P80. Red arrows indicate NLCs, suggesting a multilamellar structure. TEM images of (e) NPLLV_033, (f) NPLLV_034, and (g) NPLLV_035, made with P80, poloxamer 407, and 188, respectively. Magnification: 68,000×. Blue arrows indicate high-electron-density regions. Yellow arrows indicate vesicular structures in the lipid matrix.

TEM analysis (Figure 1 e-g) identified that all samples presented high electron density on the edges, which could indicate a lamellar structure arranged by the surfactant coating and visualized in Cryo-TEM. There are also bright vesicles in the lipid matrix, corresponding to the accommodation of the liquid lipid, which is not clear in Cryo-TEM images because samples were not contrasted with a stain. In summary, there is no visual evidence of structural differences among NLCs with the different surfactants. TEM images also suggested that the NLCs exhibit “multiple type” morphology, characterized by oil droplets dispersed within a solid lipid matrix. This observation is consistent with the literature, as formulations with a relatively high oil content (30%) tend to favor the formation of this structural arrangement.^33^ NLC multiple type (also known as type III) is advantageous when the drug has higher solubility in the liquid lipid, enhancing its entrapment in the NLC.^34^

### 3.3 Drug Release Profile

Drug release profiles were assessed to evaluate the surfactant influence in NPLLV formulations on the LV release kinetics (Figure 2). The model-independent similarity factor (f2) showed that NPLLV_033 with P80 and NPLLV_034 with P407 exhibited an *f*2 value of 73, indicating high similarity. In contrast, NPLLV_035 with P188 demonstrated lower similarity to the other two, with *f*2 values of 43 (P80) and 48 (P407), respectively. The test was performed with a dialysis membrane; therfore the release kinetics were influenced by the diffusion kinetics of LV permeation. This fact, together with the restricted number of replicates (*n* = 2), limited the usefulness of applying mathematical modeling to these release profiles. However, the comparable shape and slopes of the curves, with small standard deviations, suggest that the governing release mechanisms were similar between formulations. As mentioned before, P188 is the surfactant with the highest hydrophilicity in the study (HLB = 29), so we hypothesize that it may exhibit a stronger interaction with LV and prolong the release. This observed difference in a biorelevant medium enhances the likelihood that it can impact release in the lungs, but only an *in vivo* evaluation could confirm whether the difference would benefit therapeutic effects.

**Figure 2:**
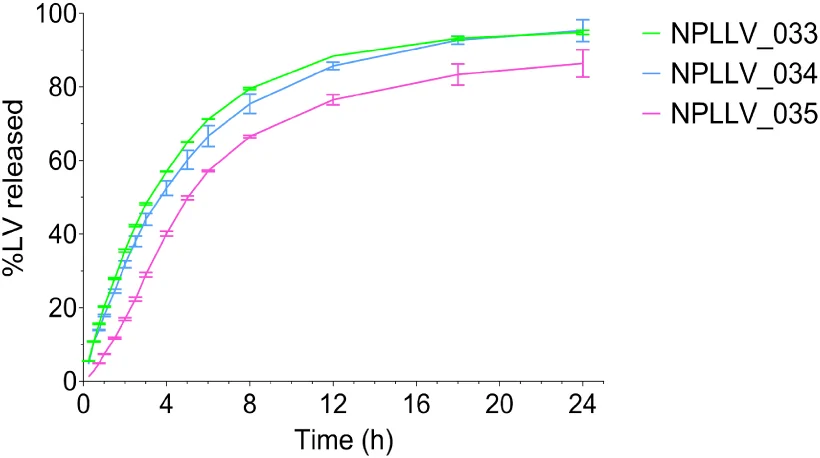
NPLLV release profiles in a Franz cell apparatus using Simulated Lung Fluid (SILF) at pH 7.4, at 37 °C under stirring at 700 rpm. NPLLV_033 with P80 (green line, *n* = 2), NPLLV_034 with P407 (blue line, *n* = 2), and NPLLV_035 with P188 (pink line, *n* = 2). The aliquots were collected and analyzed by the HPLC method.

### 3.4 Evaluation of NLC Stability

An accelerated stability test was performed (Supporting Table 1) as a screening of surfactant performance upon short-term storage. The samples were stored for 90 days at 40 °C, 75% RH, and protected from light. NLCs with both poloxamers maintained particle size distribution and zeta potential values. However, P80 coating was not enough to maintain particle size (136.3 ± 7.7 versus 303.7 ± 70.4), with a significant increment in PDI (0.227 ± 0.019 versus 0.323 ± 0.018). This behavior may be associated with the known susceptibility of P80 to auto-oxidation, which preferentially occurs at the double bonds of its fatty acid chains^35^ and can compromise nanoparticle integrity.

Impurities/degradation detected by HPLC after 90 days were not significantly different from day 1 in the poloxamer formulations, in contrast with a 10-fold increase for NLCs with P80. This degradation increment (related peak areas) was not detected before; however, the P80 was evaluated for up to 30 days under the same conditions.^14^

These results suggest that the instability of the P80 formulation involves both physical (particle growth and aggregation) and chemical (drug degradation) components. LV degradation leads to inactive molecules against bacteria, which may decrease therapeutic efficacy and facilitate bacterial resistance. So far, degradation products of LV have not been found to be toxic.^36^

### 3.5 Evaluation of Antimicrobial Activity in Planktonic Bacteria

Samples were submitted to MIC assays to evaluate their impact on the growth of bacterial strains that are relevant to lung infections (Table 4). *K. pneumoniae* strain BAA 1705 presented resistance to LV, as expected, whereas strain ATCC 700603 was susceptible and within the literature range (0.094–8 μg/mL).^37^

**Table 4:** Minimum Inhibitory Concentration (MIC) of Levofloxacin Formulations^a^

Bacterial Strain	LV (μg/mL)	NPLLV_033 (μg/mL)	NPLLV_034 (μg/mL)	NPLLV_035 (μg/mL)
*K. pneumoniae* 700603	0.78	0.78	0.78	0.78
*K. pneumoniae* BAA 1705	125	125	125	125
*S. aureus* ATCC 29213	0.19	0.19	0.19	0.19
*S. aureus* ATCC 33591	0.19	0.19	0.19	0.19
*B. cepacia* ATCC 25416	0.5	0.5	0.5	0.5
*H. influenzae* ATCC 51907	0.16	0.16	0.16	0.16

a Bacteria were treated with levofloxacin (LV) and nanoparticles with levofloxacin (NPLLV). *n* = 3 replicates, with at least two independent experiments. NPLLV_033 with P80, NPLLV_034 with P407, and NPLLV_035 with P188.

The MIC values reported for strains of *S. aureus* were among 0.5 and 4 μg/mL of LV.^40^ In our test, both strains, ATCC 29213 (methicillin-sensitive) and ATCC 33591 (methicillin-resistant) presented a MIC of 0.19 μg/mL for all treatments.

The nanoparticle placebos had no antimicrobial activity (Supporting Table 2) for these bacterial strains (except for *B. cepacia* ATCC 25416) at the tested dilutions. To our knowledge, the excipients did not present activity by themselves at the tested concentrations in planktonic bacteria. However, surfactants can sensitize membranes, which may enhance the antimicrobial efficacy of drugs or aid in biofilm disruption.^38^

There was no variation in MIC between free and nanostructured LV, indicating that nanoparticles did not enhance or decrease activity. The surfactant type had no impact on the required drug concentration to inhibit the growth of the assessed bacteria. Considering that the MIC test is a serial dilution, and each well represents a 50% dilution of the previous one, this result is coherent with the release test because 50% of the drug is released after approximately 6 h. At this time point, the bacteria are still in exponential growth and susceptible to killing before the population reaches the turbidity point.

### 3.6 Evaluation of Biofilm Inhibition: *Burkholderia cepacia*

Since the MIC test presented no differences among formulations and free LV, a biofilm inhibition test was performed (Figure 3). *B. cepacia* was selected due to its relevant role as an opportunistic pathogen that forms biofilms in cystic fibrosis lungs. This ability is directly correlated with morbidity and mortality.^39^

**Figure 3:**
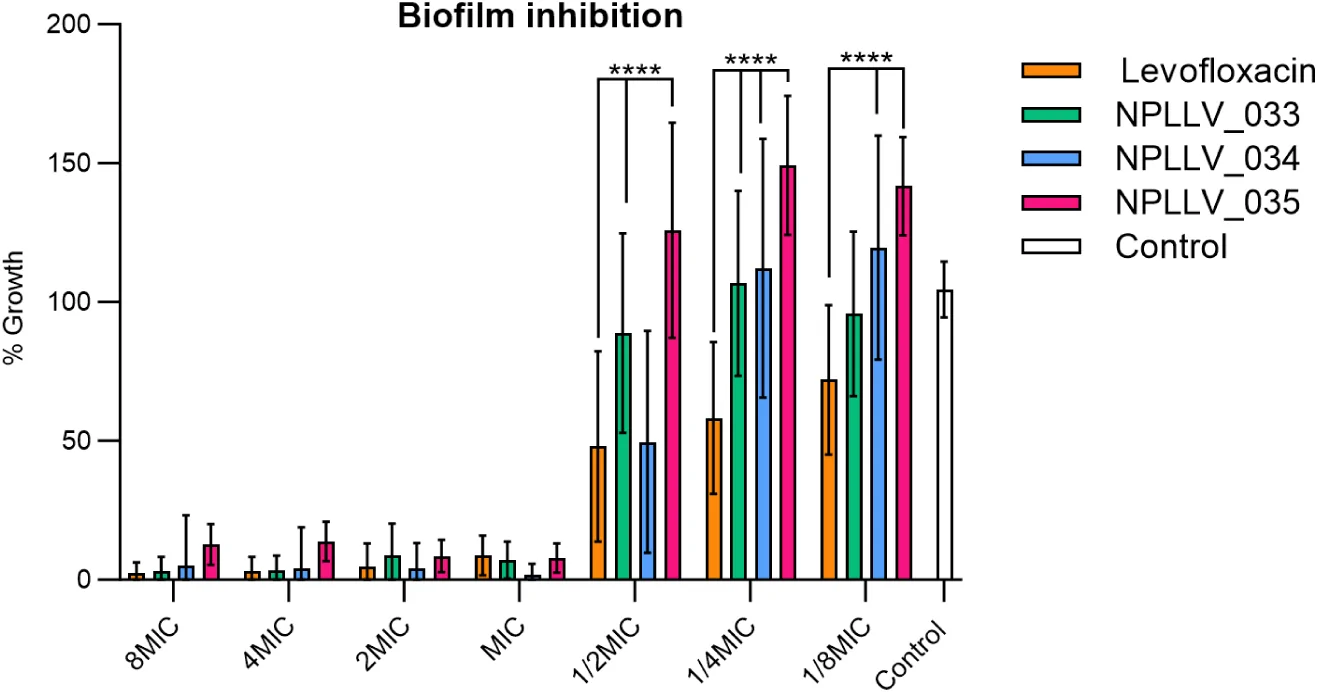
Biofilm inhibition assay using *Burkholderia cepacia* ATCC25416. Bacteria were cultured in BHI supplemented with 1% glucose for 24 h at 37 °C and 5 × 10^7^ CFU/mL. MIC = 0.5 μg/mL. Statistics: two-way ANOVA (α = 0.05), followed by Tukey’s post hoc test, was performed for comparison between each treatment and the control. Symbols *, **, and *** represent statistical significance <0.05, *p* < 0.005, and *p* < 0.0005, respectively. Nanoparticles with levofloxacin: NPLLV_033 with P80, NPLLV_034 with P407, and NPLLV_035 with P188.

Encapsulated and free LV showed full inhibitory activity at concentrations ≥MIC. Free LV significantly reduced biofilm mass at all sub-MIC concentrations tested (up to 1/8 MIC). To the best of our knowledge, this is the first report of LV efficacy against *Burkholderia* biofilms, indicating its potential effectiveness in vitro. This species has been reported to exhibit resistance to amikacin and tobramycin when used as monotherapies, often requiring combination therapy for effective treatment, which is why this finding is relevant to infection therapy.^40^

The NPLLV_034 formulation containing P407 reduced biofilm mass up to 1/2MIC, whereas the other nanoparticles did not. These results suggest that the higher bacterial load in this assay, combined with glucose supplementation, may have required a greater amount of LV to be released in the initial hours compared to the MIC assay, which may explain the differences above the MIC. Some studies suggest that sustained release ensures that bacteria are exposed to effective drug concentrations throughout their growth cycle, which is crucial for eradicating biofilms resistant to short-term treatments.^41^

### 3.7 Evaluation of Biocompatibility by *In Vitro* Cell Viability of Lung Cells

The Calu-3 cell line was chosen because it is widely used as an in vitro model for lung/nasal epithelium to evaluate drug cytotoxicity and transepithelial transport.^29,45^ The tested concentrations were based on a previous study with A549 lung cells exposed to free or nanoencapsulated LV. When related to the MIC determined for *Klebsiella pneumoniae* ATCC 700603 (0.78 μg/mL)^2^, values are equivalent to 1.3, 13, and 130 times the determined MIC.

Samples were incubated with cells for 24 h treatment and evaluated by the MTT assay (Figure 4). Free LV maintained the viability of Calu-3 cells at all tested concentrations, in agreement with previous results in A549 cells^2^. When LV was loaded into nanoparticles at the highest tested concentration, they reduced cell viability to less than 50% (100 μg/mL, 50-fold dilution). The same dilution of placebo nanoparticles also reduced viability, although to a minor extent, indicating that the carrier itself reduces cell viability.

**Figure 4:**
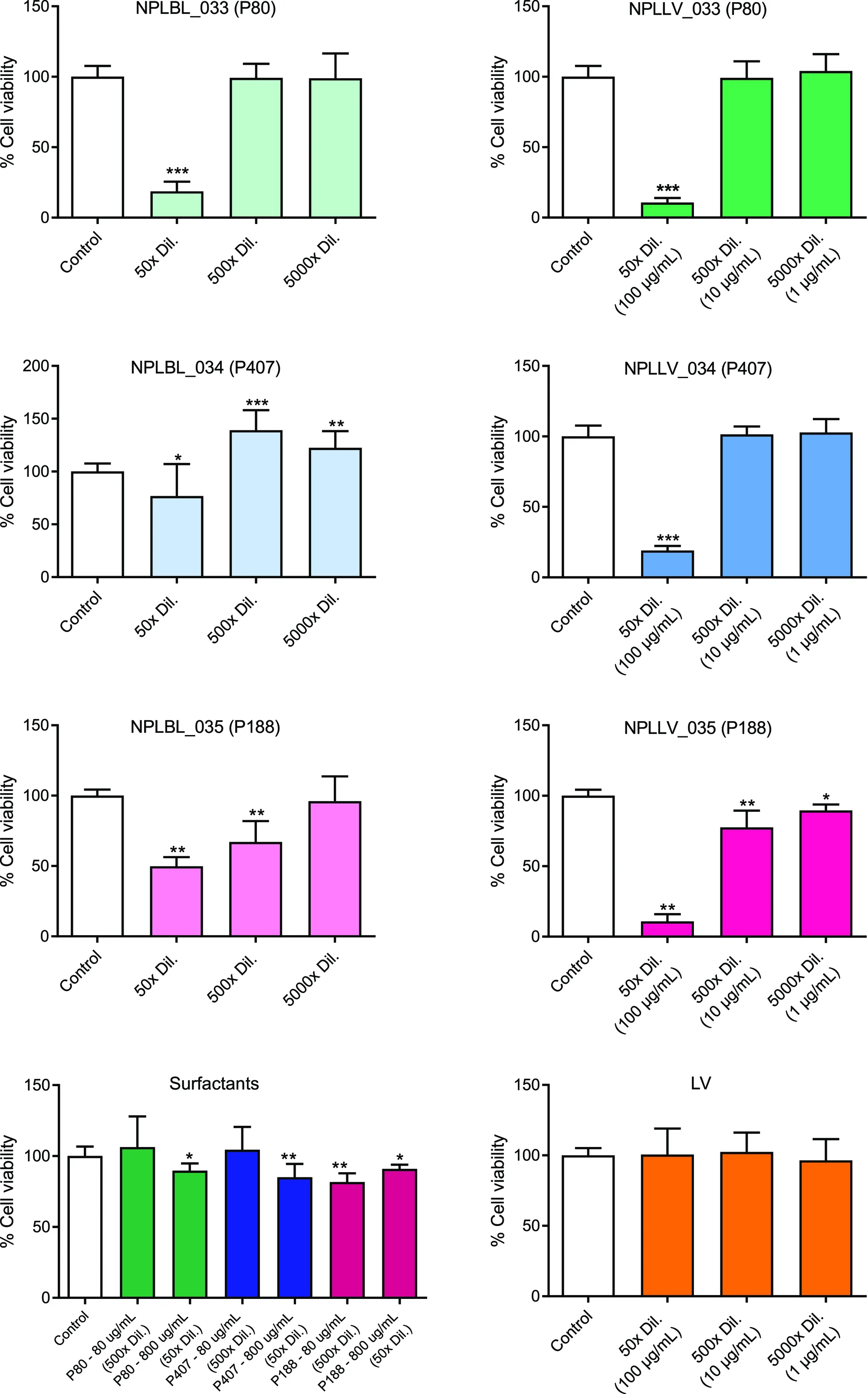
Cell viability assay (MTT assay) of the nondifferentiated Calu-3 cell line. Cell viability (%) was evaluated after 24 h of treatment with samples in 96-well microplates (5 × 10^4^ cells/well). Positive viability control: DMEM medium (white bars). Samples: blank (NPLBL) and LV-loaded nanoparticles (NPLLV) prepared with P80 (green bars), P407 (blue bars), and P188 (pink bars); LV aqueous solution (orange bars); aqueous solutions of the surfactants (green, purple, and red bars). Results are expressed as mean ± standard deviation, obtained from at least 2 independent assays, with *n* ≥ 3 replicates per assay. Symbols *, **, and *** represent statistical significance compared to the viable cell control (*p* < 0.05, *p* < 0.005, and *p* < 0.0005, respectively), determined by the nonparametric Mann–Whitney test.

This effect may be associated with the high concentration of nanoparticles in the formulation.^42^ Noteworthy, osmolarity measurements performed with the NPLLV_034 formulation indicated a hypo-osmotic system (86 mOsm/kg). In addition, all samples were diluted at least 50-fold in cell culture medium prior to testing, suggesting that osmotic effects are unlikely to be the main drivers of the observed cytotoxicity. Overall, these results suggest that the observed cytotoxicity is primarily related to formulation components, particularly the surfactant type, rather than osmotic effects.

At 10 μg/mL of LV (500-fold dilution), the nanoparticles maintained cell viability, except for NPLV_035 with P188 (<80% cell viability). The same patterns were observed for the placebo counterparts and pure surfactants, as the P188 solution was the only one to significantly decrease cell viability at the same dilution (500-fold). Noteworthy, we did not detect chemical interference of surfactants with MTT.

These findings point to a negative effect of P188 on Calu-3 cell viability under the tested conditions. In contrast, P407 was associated with higher cell viability, as also noticed by Allotey-Babingtonet et al. in specific types of cells (rat liver epithelial cells (WB), murine kidney cells (MDCK), and triple-negative human breast cancer cells (MDA-MB231).^42^

To confirm the effect on Calu-3 cell viability, a neutral red assay was performed (Supporting Figure 3), which reflects cell membrane integrity instead of mitochondrial activity (MTT). Free LV did not interfere with Calu-3 cell viability, while all NLCs significantly reduced cell viability at 100 μg/mL of LV (50-fold dilution, *p* < 0.0005), as observed with MTT assays. At 10 μg/mL (500-fold dilution), cell viability was maintained for all formulations, whereas MTT indicated reduced metabolic activity for NPLLV_035­(P188). This suggests that P188 may induce early metabolic alteration without causing detectable lysosomal damage within 24 h.

To further explore concentration-dependent effects, additional LV concentrations (50, 25, and 10 μg/mL) were tested (Figure 5), and the incubation time was extended to 48 h, considering the approximate doubling time of Calu-3 cells (∼40 h).^43^

**Figure 5:**
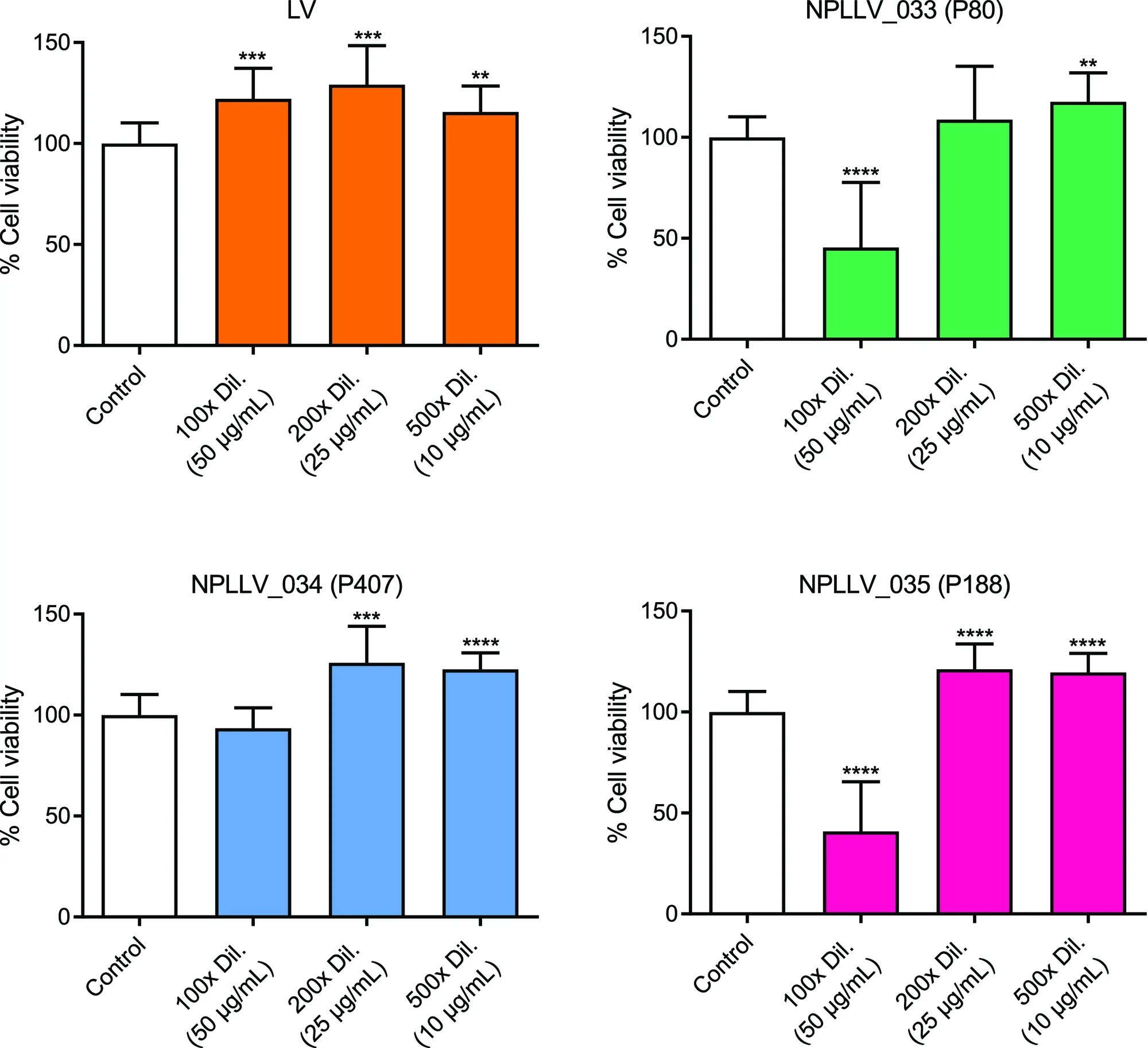
Cell viability (Neutral Red assay) of nondifferentiated Calu-3 cell line. Cell viability (%) was evaluated after 48 h of treatment with samples in 96-well microplates (5 × 10^4^ cells/well). Samples: LV-loaded NLCs prepared with the three surfactants (NPLLV_033 with P80 (green bars), NPLLV_034 with P407 (blue bars), and NPLLV_035 with P188 (pink bars), and LV aqueous solution (orange bars) in three different concentrations (related to LV): 50, 25, or 10 μg/mL. All treatments were compared with the positive control (DMEM medium, white bars). Cell viability (%) was evaluated by the Neutral Red assay in 96-well microplates with a density of 5 × 10^4^ cells/well, stimulated for 48 h. Results are expressed as mean ± standard deviation, obtained from 3 independent assays, with *n* ≥ 3 replicates per assay. Symbols **, ***, and **** represent statistical significance compared to the viable cell control (*p* < 0.005, *p* < 0.0005, and *p* < 0.00005, respectively), determined by the nonparametric Mann–Whitney test.

Under these conditions, LV and NPLLV_034 (P407) did not inhibit cell proliferation at any tested concentration. In contrast, NPLLV_033 and NPLLV_035 reduced cell viability at the highest concentration (50 μg/mL). The longer incubation time probably facilitated the detection of lysosomal damage induced by the P188 and P80 formulations. Overall, lower concentrations of the nanodispersions did not exhibit cytotoxic effects in Calu-3 cells. These results supported subsequent viability assessment in differentiated Calu-3 cells and guided the selection of NPLLV_034 as the NLC formulation for further experiments.

### 3.8 Cell Viability in Differentiated Calu-3 Cells

Cell viability assay on differentiated Calu-3 cells was conducted, as their polarization may change their characteristics and alter their tolerance to different stimuli. The neutral red assay with NPLLV_034 at 50 μg/mL showed 71 ± 6% cell viability (data not shown), a value considered weakly cytotoxic,^44^ but sufficient to alter inflammatory responses, besides denoting a higher susceptibility of differentiated cells. Therefore, we set the LV concentration at 25 μg/mL for further IL-8 assessments.

Figure 6 shows that 25 μg/mL of LV was a safe concentration for differentiated Calu-3 cells treated for 48 h, and LPS 1 μg/mL was not considered cytotoxic to the cells. However, TEER measurements after 48 h-stimuli exposition showed a reduction in the values (from ∼792 Ω.cm^2^ to ∼557 Ω.cm^2^). This reduction did not compromise the integrity of the cell monolayer, as TEER values were >500 Ω.cm^2^.^23^ Also, this value is above the cutoff (450) related to the increase in fluorescein-sodium permeability, indicating that this value would likely not be sufficient to cause increments in LV absorption.^45^

**Figure 6:**
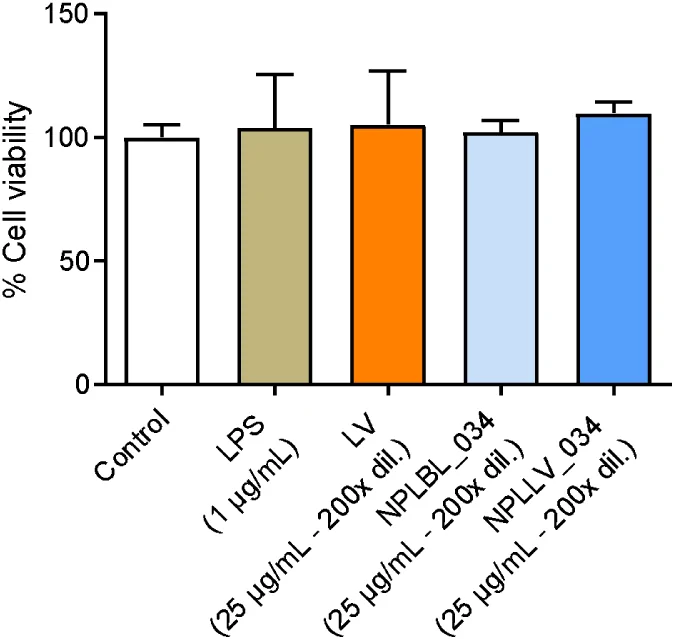
Cell viability after 48 h-exposure to NLCs, expressed as cell membrane integrity by neutral red evaluation in the differentiated Calu-3 cell line. The graph corresponds to the percentage of Calu-3 cell viability after treatment with lipopolysaccharide (LPS) at 1 μg/mL (beige bar); LV aqueous solution (orange bar); NPLBL_034 (light blue bar); and NPLLV_034 (blue bar) at a 25 μg/mL concentration. All treatments were compared with the positive control (DMEM medium, white bar). Cell viability (%) was evaluated by the neutral red assay in transwell inserts (6.5 mm, 0.4 μm pore diameter size, polyester membrane, 0.33 cm^2^ effective area for growth), in 24-well microplates with an initial density of 5 × 10^5^ cells/well, stimulated for 48 h. Results are expressed as mean ± standard deviation, obtained from 2 independent assays, with *n* = 2 replicates per assay. There was no statistical significance between treatments and the control (nonparametric Mann–Whitney test).

### 3.9 IL-8 Cytokine Secretion Profile from Calu-3 Differentiated Cells

We evaluated the effect of nanoparticle formulations on differentiated Calu-3 cells by measuring IL-8 secretion as a marker of epithelial inflammation (Figure 7).^46^ Higher cytokine levels were detected in the apical compartments, but it has a smaller volume (approximately 100 μL), and the presence of mucus may concentrate secreted factors. The basolateral compartment has a larger volume (10-fold), leading to dilution effects. Additionally, epithelial polarization may contribute to differential IL-8 secretion across compartments. The potential anti-inflammatory effect of LV was previously reported, with reductions in IL-8 and IL-6 secretions at LV concentrations above 100 μg/mL. At lower concentrations (10–30 μg/mL), LV did not significantly reduce cytokine secretion in NL20 cells,^47^ a trend observed in Calu-3 cells.

**Figure 7:**
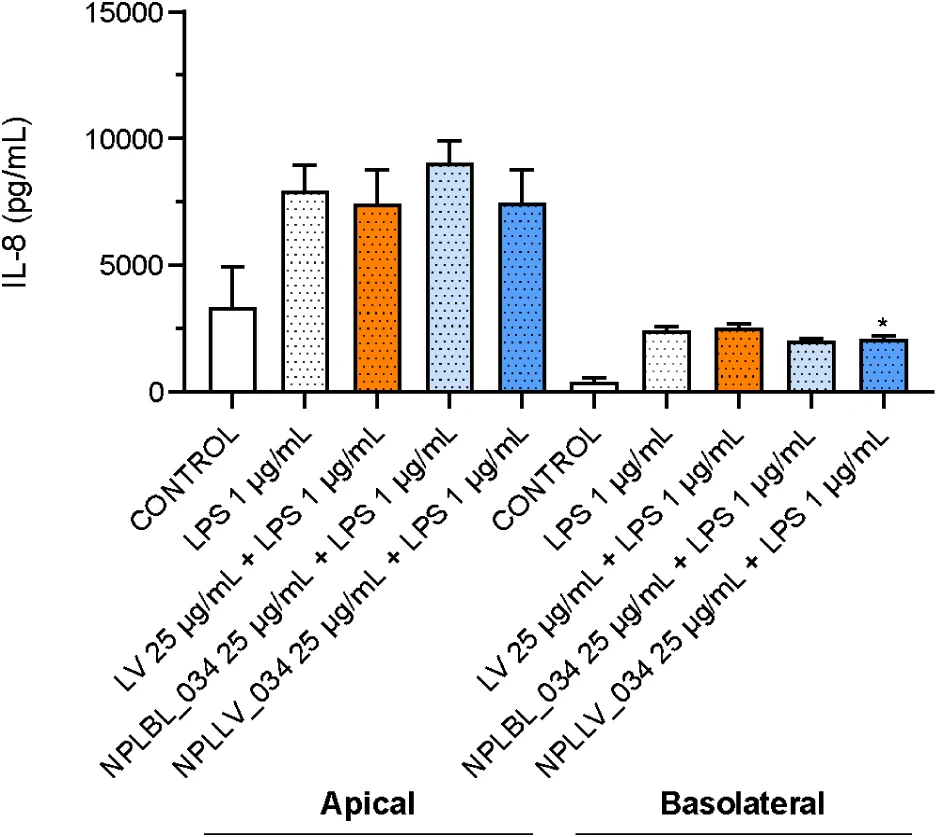
IL-8 cytokine secretion from differentiated Calu-3 cells. Differentiated Calu-3 cells were treated with stimuli after 14 ± 2 days of differentiation, and TEER was measured. Stimuli (LV solution, NPLBL_034, and NPLLV_034 at 25 μg/mL), with or without LPS at 1 μg/mL, were plated on the apical compartment of the transwell (*n* = 2, two independent experiments) for 48 h. Controls: cell culture medium and LPS at 1 μg/mL, were used as parameters for basal and stimulated IL-8 secretion from Calu-3.

Regardless of the compartment, stimulation with LPS (1 μg/mL) increased IL-8 secretion compared to the controls. Neither LV nor NLC formulations reversed or exacerbated this response. Interestingly, NPLLV_034 at 25 μg/mL of LV produced a statistically significant reduction in LPS-induced IL-8 secretion compared to free LV at the same concentration (*p* < 0.05). However, the magnitude of this reduction was small and likely not biologically relevant when compared to the LPS-treated group.

Jackson et al. reported that P407 can prevent plasma protein adsorption onto microsphere surfaces and limit the opsonization-mediated activation of neutrophils, suggesting a potential anti-inflammatory effect of P407.^48^ Although evidence regarding P407 and epithelial cytokine release remains limited, its ability to reduce protein adsorption may contribute to the lack of exacerbated IL-8 secretion observed in our system.

### 3.10 Nanoparticle Integrity Post-Nebulization

Since NPLLV_034 showed the best performance, it was selected for a preliminary feasibility evaluation upon nebulization. The P407 formulation was nebulized using a portable mesh nebulizer . A total of 93% of the formulation was recovered, which was considered high for a nonsealed system (Supporting Figure 5). The nebulized formulation presented a similar visual appearance to the nonnebulized formulation. Additionally, size, PDI, and zeta potential were assessed before and after nebulization, and no significant differences were observed (Supporting Figure 5).

These results indicate that the formulation maintained its physical properties after nebulization under the tested conditions. Further studies will assess drug loading, release behavior, and aerosol performance to confirm its suitability for pulmonary delivery.

## 4 Conclusion

Nanostructured lipid carriers loaded with levofloxacin were prepared with three non ionic surfactant coatings, two of them for the first time (P188, P407), and comparatively evaluated regarding their effects on physicochemical and biological outcomes. They presented similar particle size ranges, zeta potential, and high LV loading when freshly prepared, but the P80-coated NLCs did not preserve LV stability or particle size distribution under accelerated stability conditions. LV maintained the antimicrobial activity profile regardless of encapsulation, whereas entrapment reduced biofilm inhibition at sub-MIC doses. Importantly, LV showed great potential for inhibition of *Burkholderia* biofilms, a relevant finding, and P407 was the most promising coating. In Calu-3 cells, both blank and LV-loaded NLCs were cytotoxic at a 50-fold dilution (100 μg/mL LV), with differentiated cells being more sensitive; notably, only P407-coated NLCs were able to maintain cell viability at 25 μg/mL. IL-8 secretion assays demonstrated that P407-coated NLCs did not exacerbate LPS-induced inflammatory conditions, supporting further investigation of these systems as antibiotic carriers. The nanoparticles also remained stable after nebulization, with no evidence of agglomeration, demonstrating that the physicochemical characteristics evaluated (particle size, PDI, and zeta potential) were preserved after nebulization under the tested conditions. Further evaluation should be performed to evaluate drug stability and release profile after nebulization. Therefore, we demonstrated that the surfactant coating can impact LV activity and cytotoxicity, adding knowledge to support formulation decisions for the inhalation route. In particular, P407-coated NLCs could effectively improve LV stability under accelerated storage conditions, preserve antimicrobial activity, maintain epithelial cell viability in vitro, and not exacerbate inflammatory responses under LPS-induced inflammation. These results highlight this system as a promising candidate for further in vivo studies to assess its pharmacokinetics and therapeutic efficacy against infections.

## Supplementary Material



## Notes

Supporting Figure 1: particle size distribution of NLCs measured by Dynamic Light Scattering (DLS), with data measured by intensity. Supporting Figure 2: particle size distribution of NLCs measured by Nanotracking Analysis (NTA). Supporting Table 1: physicochemical characterization of NLCs’ stability studies. Supporting Figure 3: cell viability (Neutral Red Assay) of nondifferentiated Calu-3 cell line . Supporting Figure 4: characterization of differentiated Calu-3. Supporting Figure 5: particle size distribution of NPLLV_034 with P407, represented by number, before and after nebulization (A); nebulization setup (B). Supporting Table 2: Minimum Inhibitory Concentration (MIC) of placebo formulations and viability control (PDF)
