# Supplementary material for: The Impact of Surfactants in Tailoring Nanostructured Lipid Carriers for Inhaled Levofloxacin
Source: ACS Omega. 2026 Aug 13;11(33):49255–67. doi: 10.1021/acsomega.6c01967 (PMC13520480; doi:10.1021/acsomega.6c01967)
Supplement: Supplementary file 1 [file ao6c01967_si_001.pdf]

Supporting information

The Impact of Surfactants in Tailoring Nanostructured Lipid Carriers for Inhaled Levofloxacin

Viviane Lucia Beraldo de Araujo [a,b *], Samira Elisa Alves Geraldo [b,c], Marcelo van Vliet Lima [b,d], Gabriel da Silva Cordeiro [b], João Paulo Guarnieri [b], Marcelo Lancellotti [b], Karina Cogo-Müller [b], Catarina Raposo [b], Laura Oliveira-Nascimento [b]

[a] School of Chemical Engineering, Universidade Estadual de Campinas, Campinas, Brazil.

[b] Faculty of Pharmaceutical Sciences, Universidade Estadual de Campinas, Campinas, Brazil.

[c] School of Medical Sciences, Universidade Estadual de Campinas, Campinas, Brazil.

[d] Sanofi Medley Farmacêutica Ltda, Campinas, Brazil.

* Corresponding author: vivi.beraldo@gmail.com

Supporting Figure 1. Particle size distribution of NLCs measured by Dynamic Light Scattering (DLS).

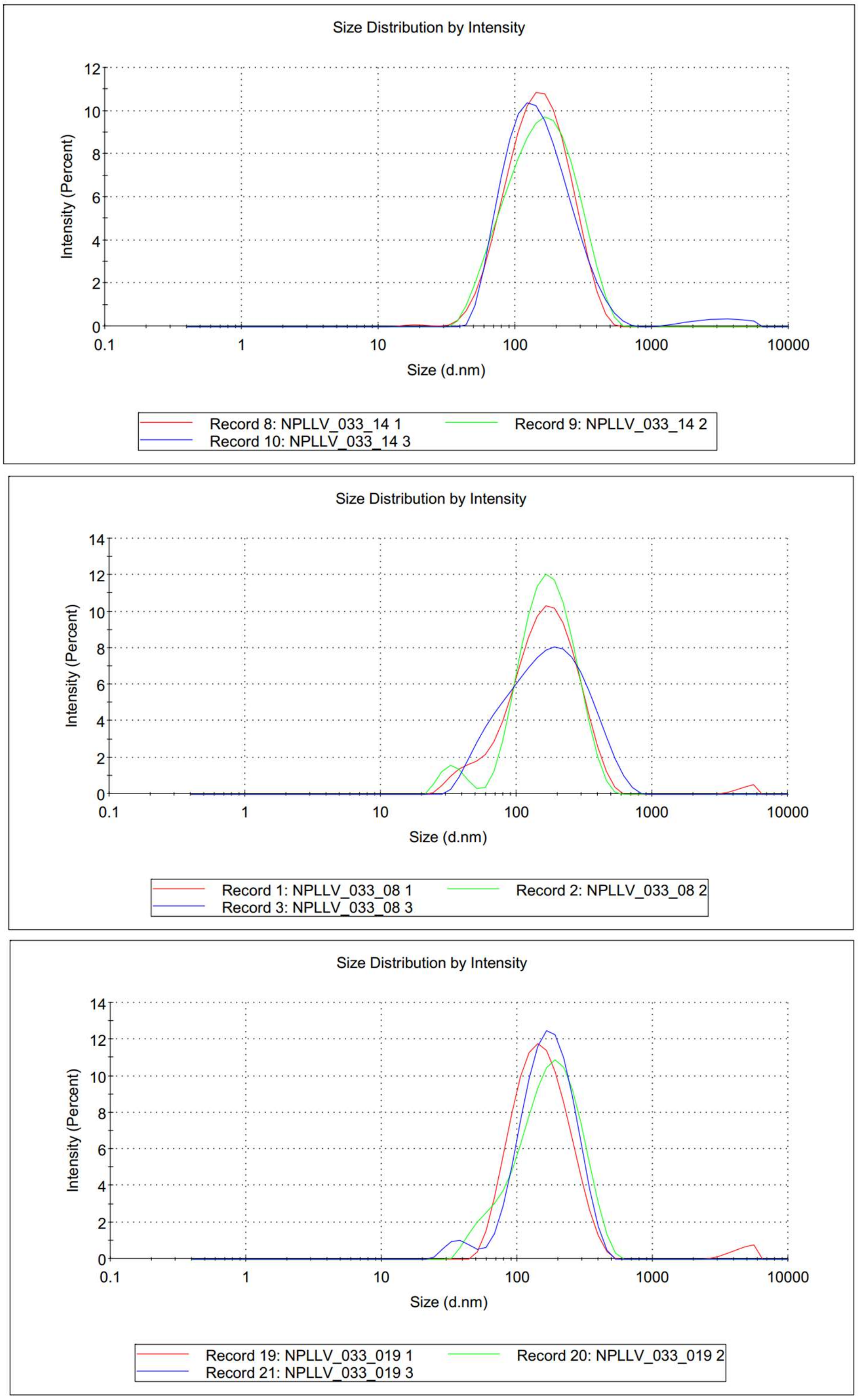

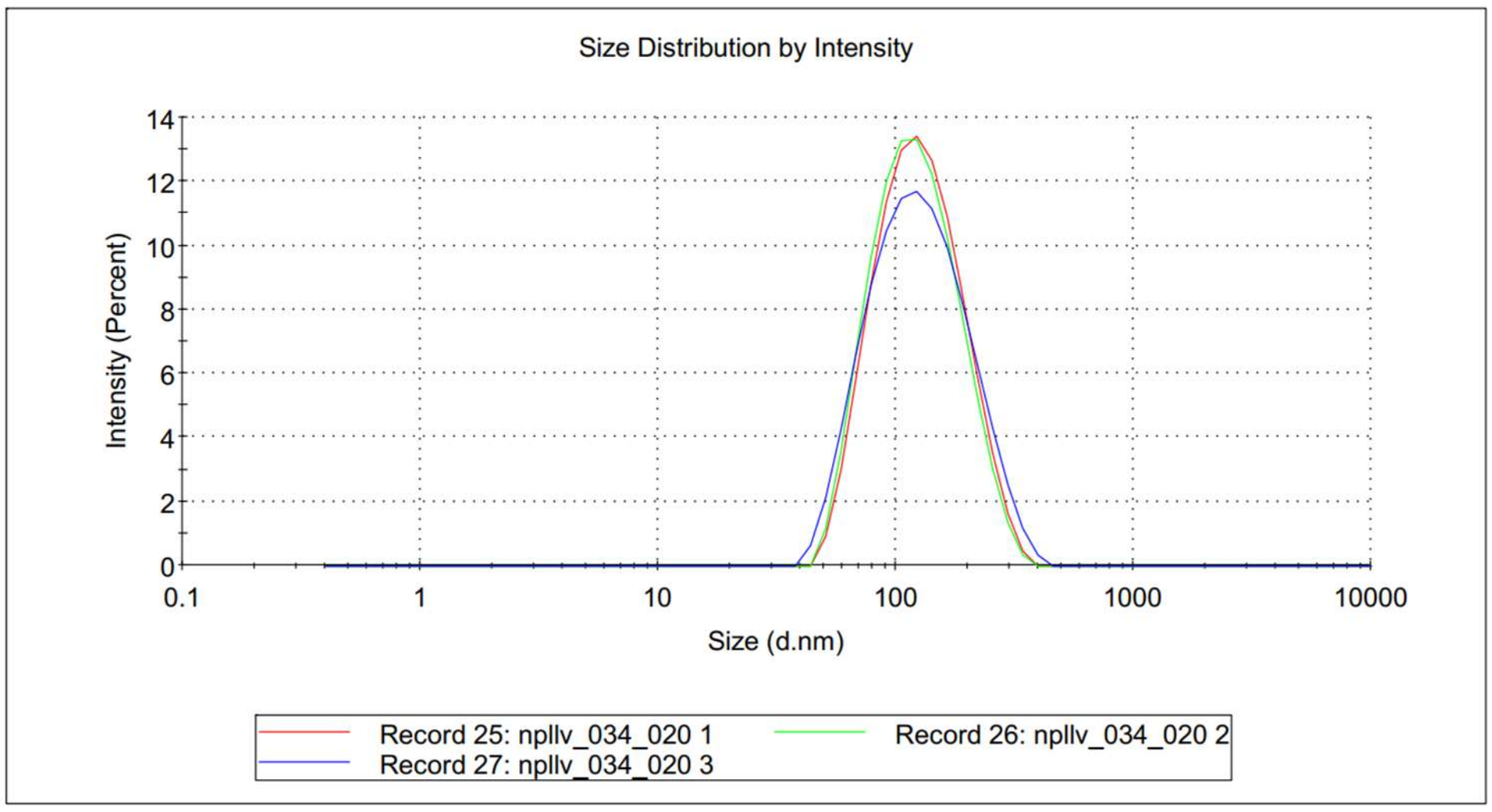
Size Distribution by Intensity
Intensity (Percent)
Size (d.nm)
0
2
4
6
8
10
12
14
0.1
1
10
100
1000
10000
Record 25: npllv_034_020 1
Record 26: npllv_034_020 2
Record 27: npllv_034_020 3

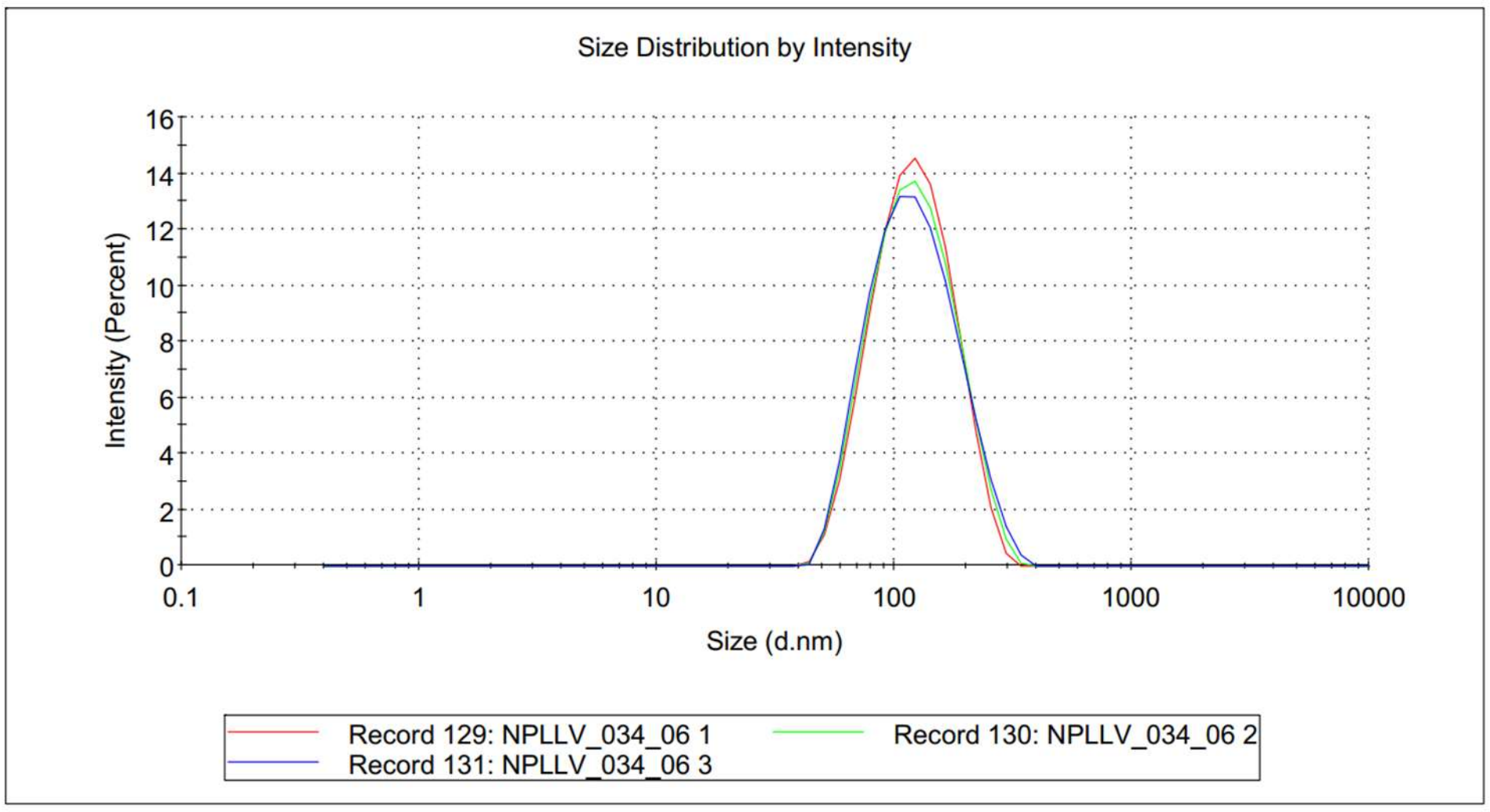
Size Distribution by Intensity
Intensity (Percent)
Size (d.nm)
0
2
4
6
8
10
12
14
16
0.1
1
10
100
1000
10000
Record 129: NPLLV_034_06 1
Record 130: NPLLV_034_06 2
Record 131: NPLLV_034_06 3

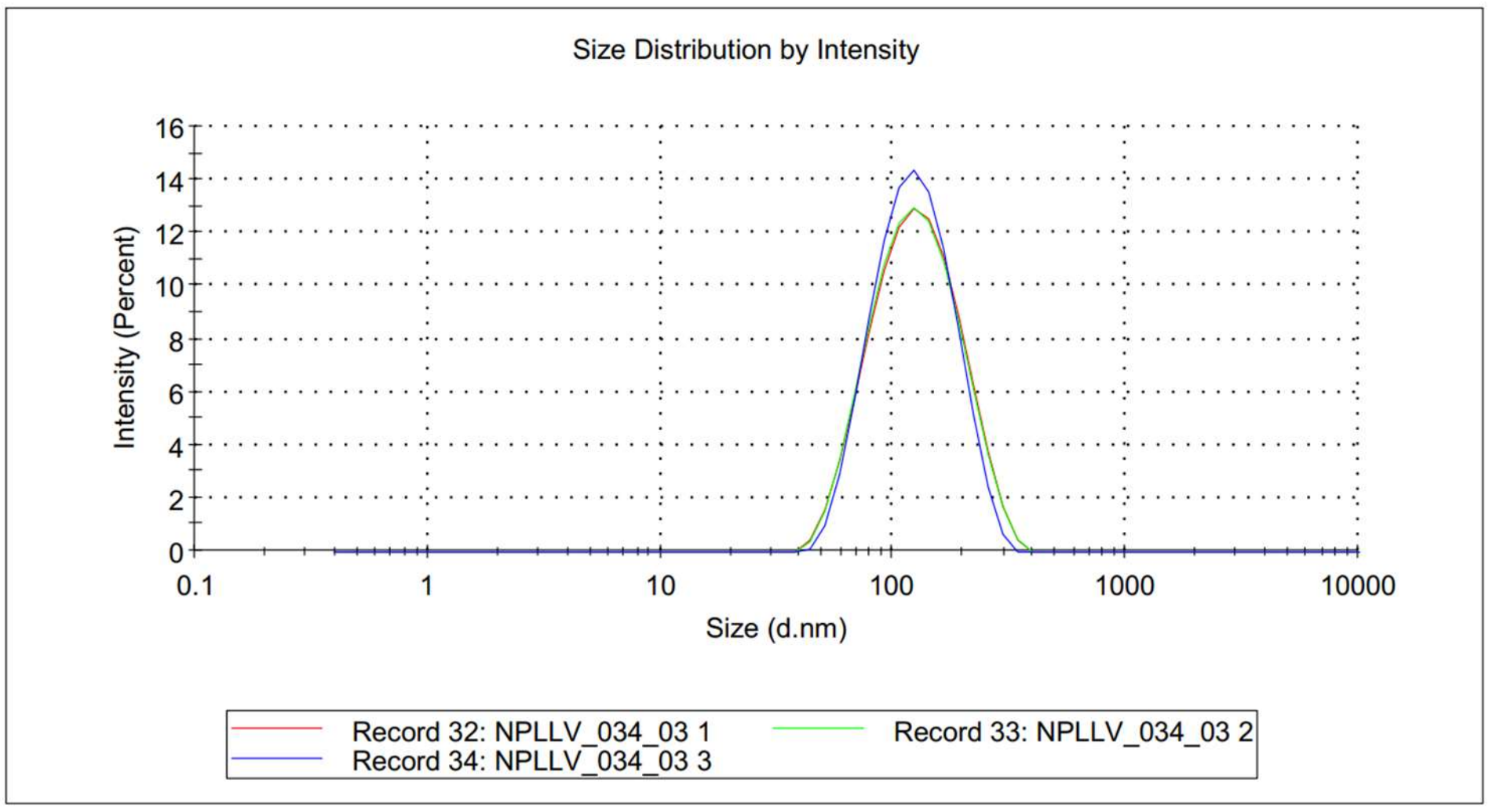
Size Distribution by Intensity
Intensity (Percent)
Size (d.nm)
0
2
4
6
8
10
12
14
16
0.1
1
10
100
1000
10000
Record 32: NPLLV_034_03 1
Record 33: NPLLV_034_03 2
Record 34: NPLLV_034_03 3

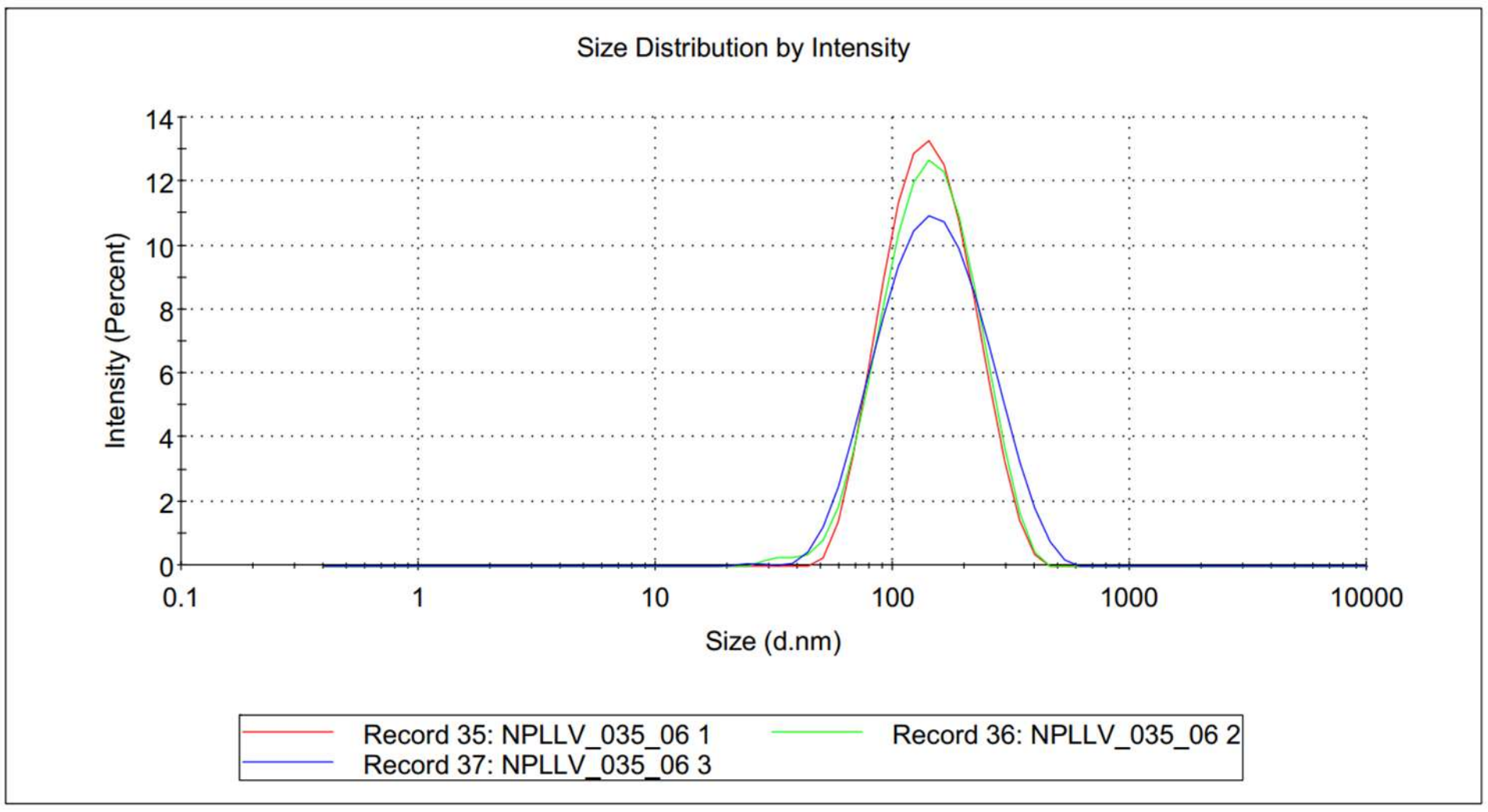
Size Distribution by Intensity
Intensity (Percent)
14
12
10
8
6
4
2
0
0.1
1
10
100
1000
10000
Size (d.nm)
Record 35: NPLLV_035_06 1
Record 36: NPLLV_035_06 2
Record 37: NPLLV_035_06 3

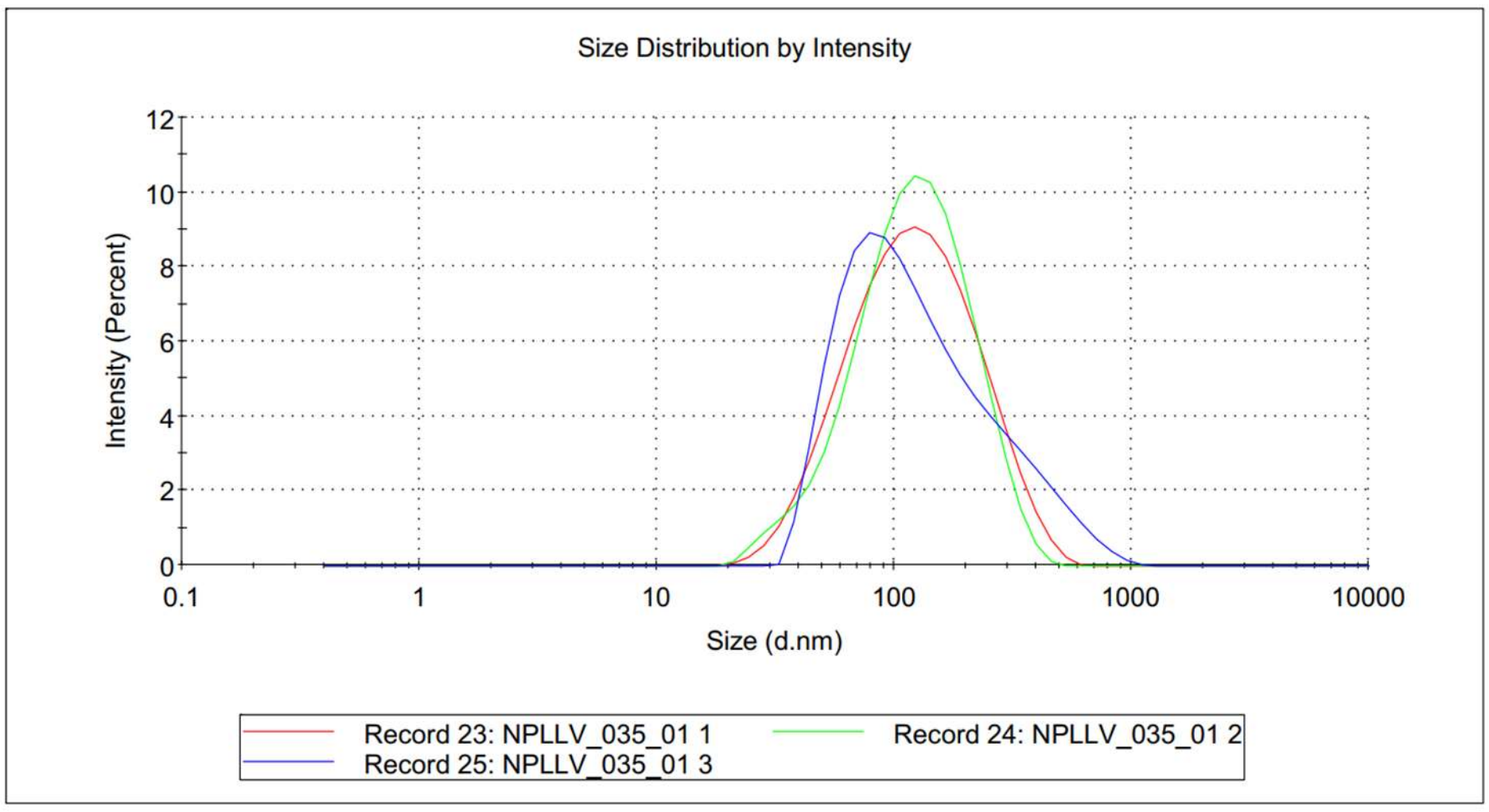
Size Distribution by Intensity
Intensity (Percent)
12
10
8
6
4
2
0
0.1
1
10
100
1000
10000
Size (d.nm)
Record 23: NPLLV_035_01 1
Record 24: NPLLV_035_01 2
Record 25: NPLLV_035_01 3

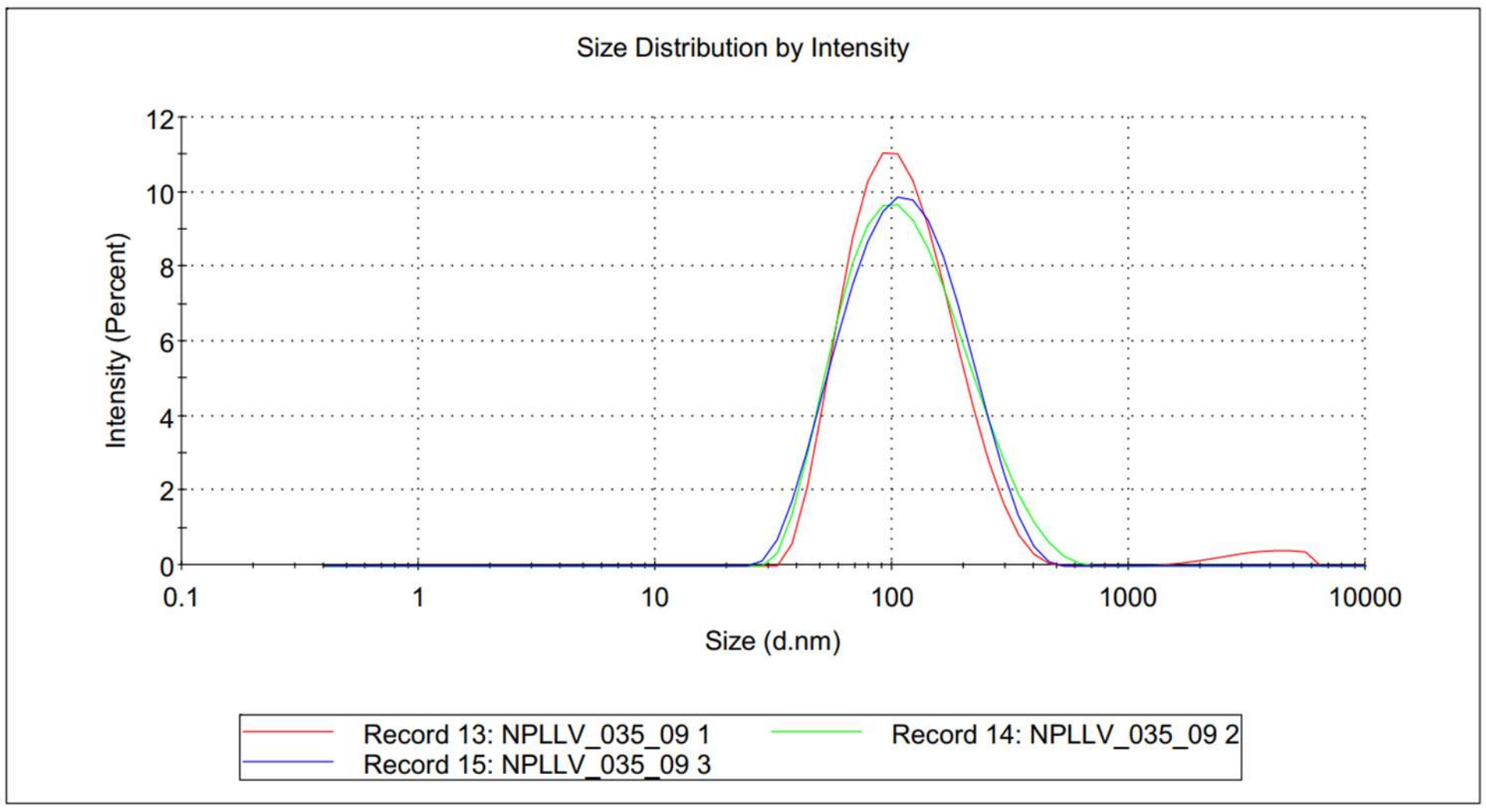
Size Distribution by Intensity
Intensity (Percent)
12
10
8
6
4
2
0
0.1
1
10
100
1000
10000
Size (d.nm)
Record 13: NPLLV_035_09 1
Record 14: NPLLV_035_09 2
Record 15: NPLLV_035_09 3

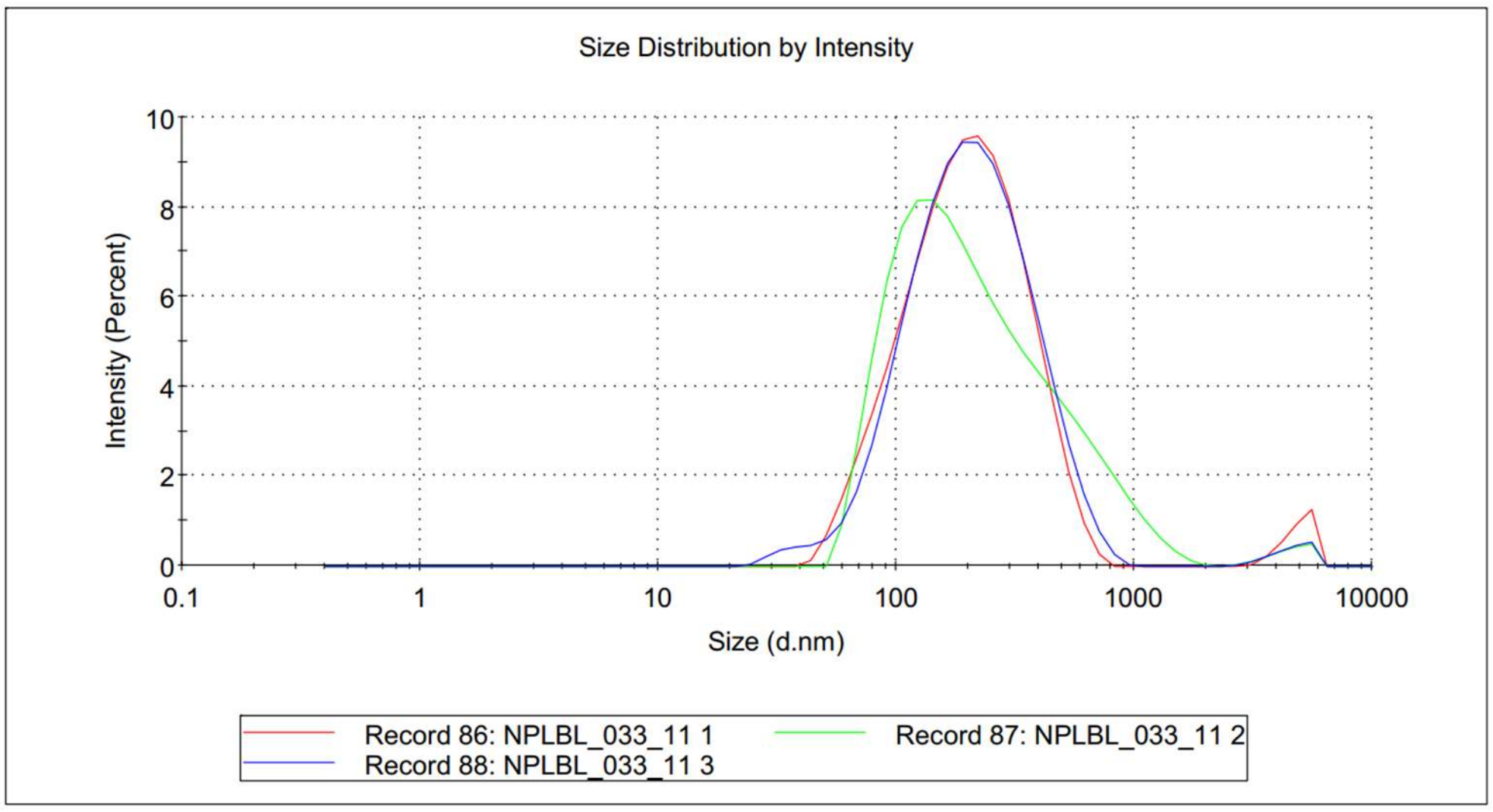
Size Distribution by Intensity
Intensity (Percent)
10
8
6
4
2
0
0.1
1
10
100
1000
10000
Size (d.nm)
Record 86: NPLBL_033_11 1
Record 87: NPLBL_033_11 2
Record 88: NPLBL_033_11 3

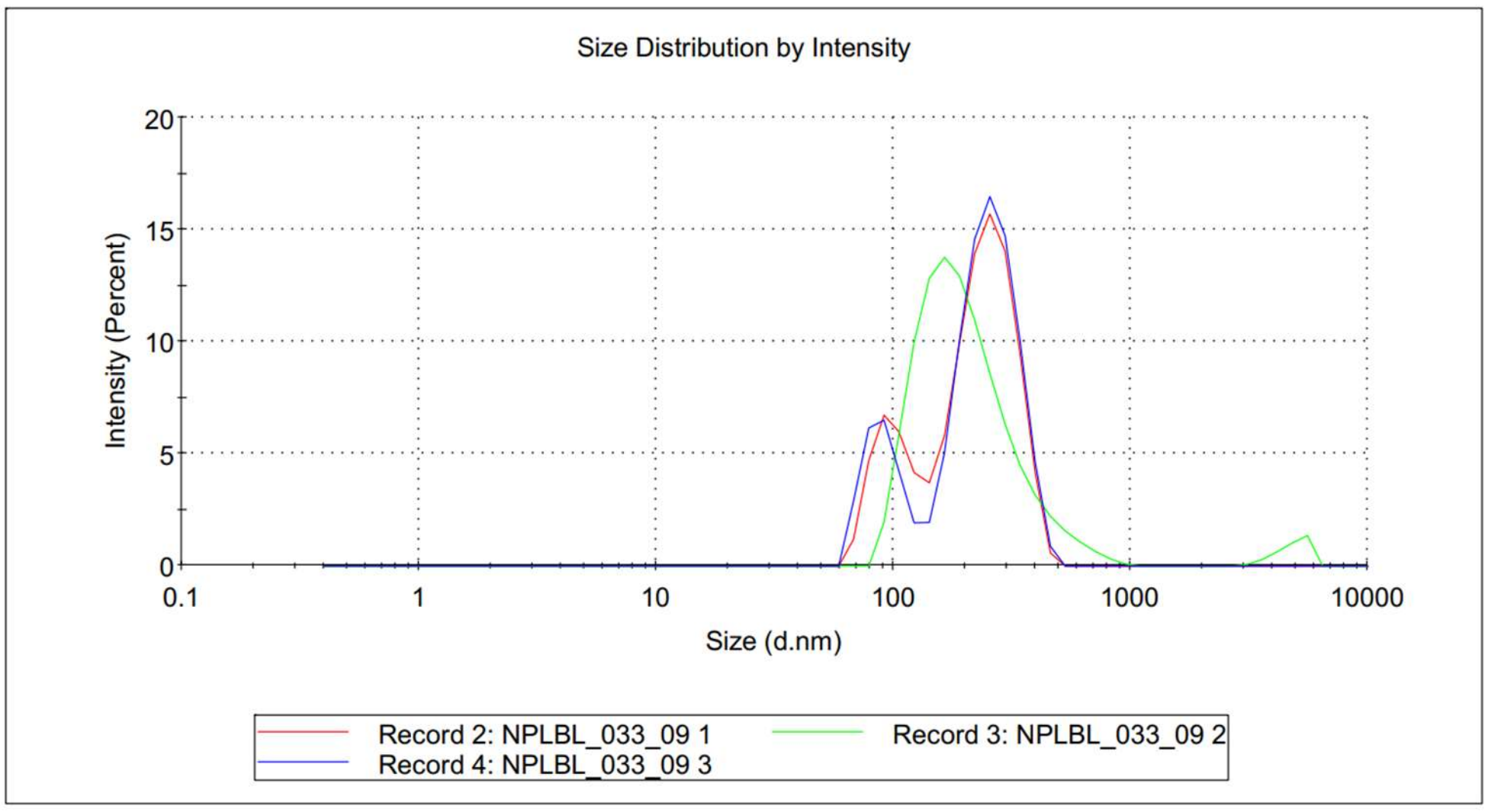
Size Distribution by Intensity
Intensity (Percent)
20
15
10
5
0
0.1
1
10
100
1000
10000
Size (d.nm)
Record 2: NPLBL_033_09 1
Record 3: NPLBL_033_09 2
Record 4: NPLBL_033_09 3

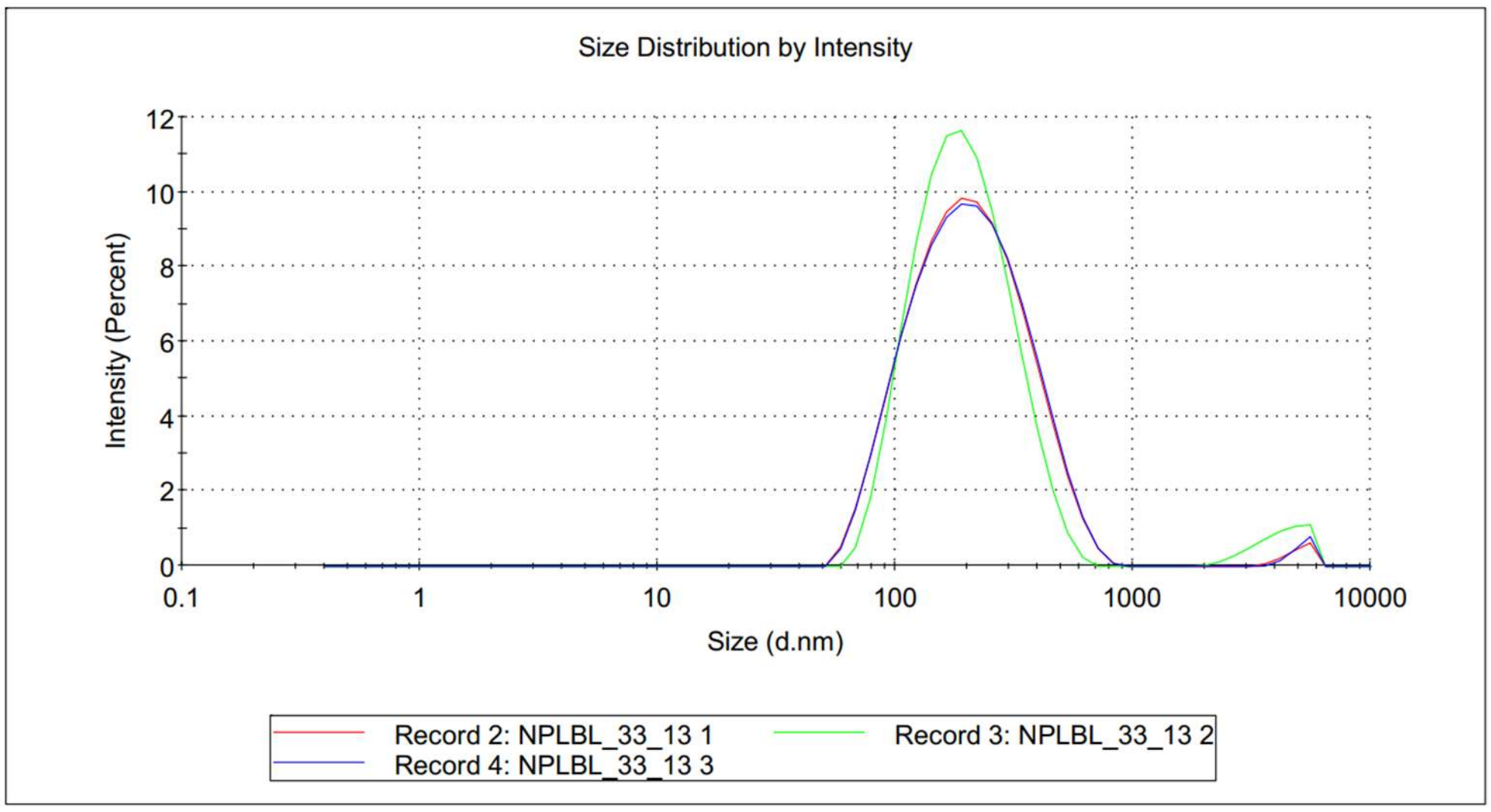
Size Distribution by Intensity
Intensity (Percent)
12
10
8
6
4
2
0
0.1
1
10
100
1000
10000
Size (d.nm)
Record 2: NPLBL_33_13 1
Record 3: NPLBL_33_13 2
Record 4: NPLBL_33_13 3

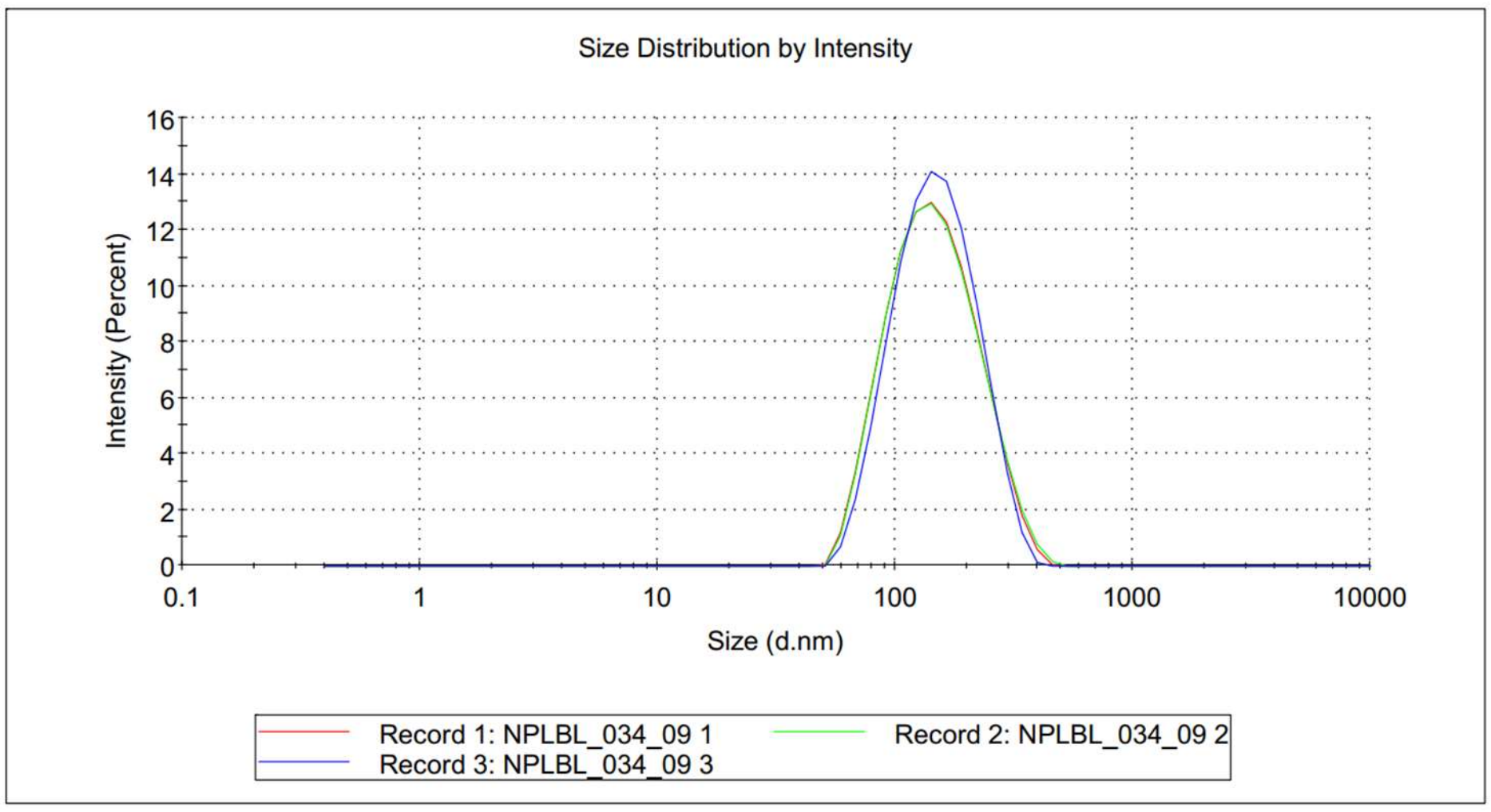
Size Distribution by Intensity
Intensity (Percent)
Size (d.nm)
Record 1: NPLBL_034_09 1
Record 2: NPLBL_034_09 2
Record 3: NPLBL_034_09 3

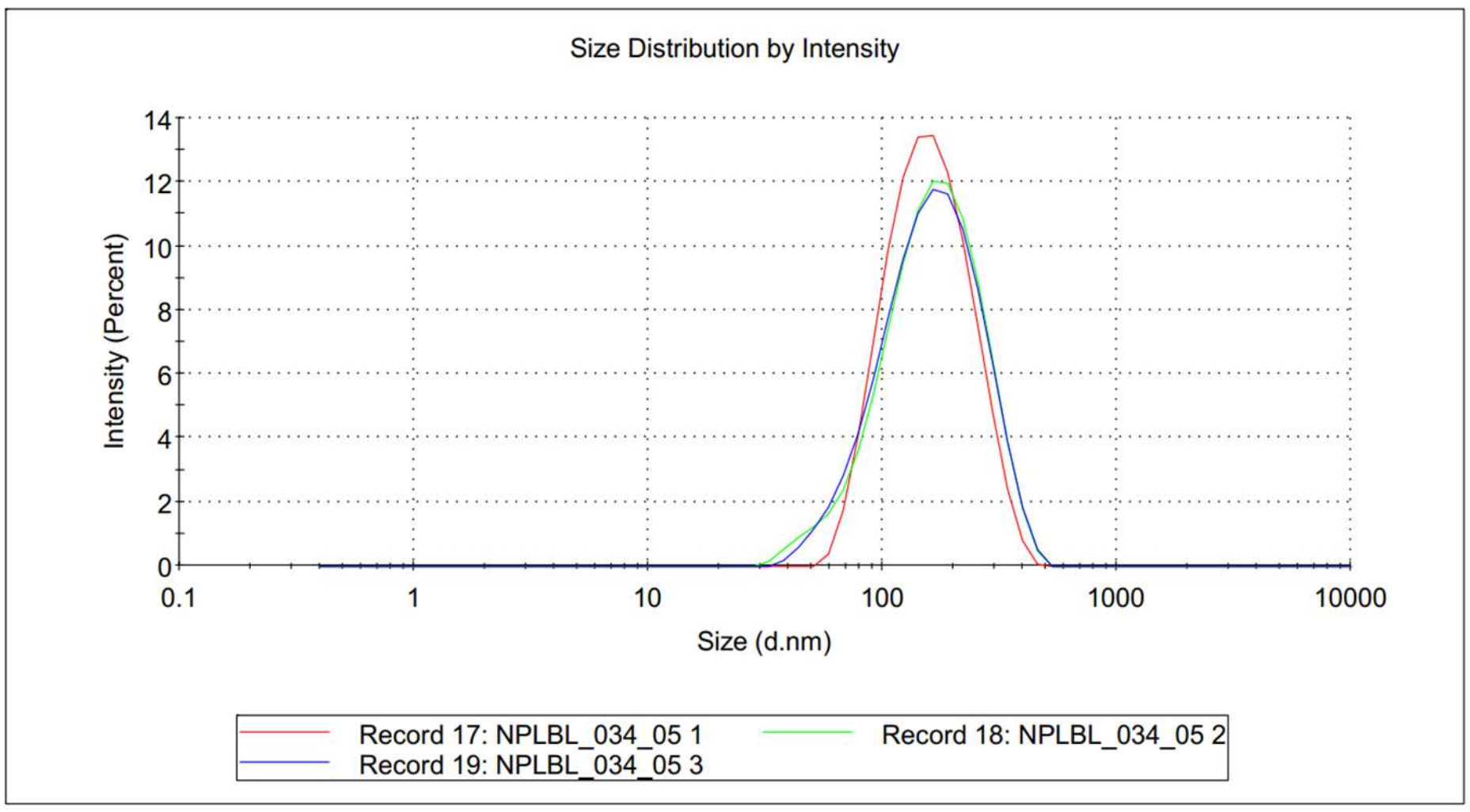
Size Distribution by Intensity
Intensity (Percent)
Size (d.nm)
Record 17: NPLBL_034_05 1
Record 18: NPLBL_034_05 2
Record 19: NPLBL_034_05 3

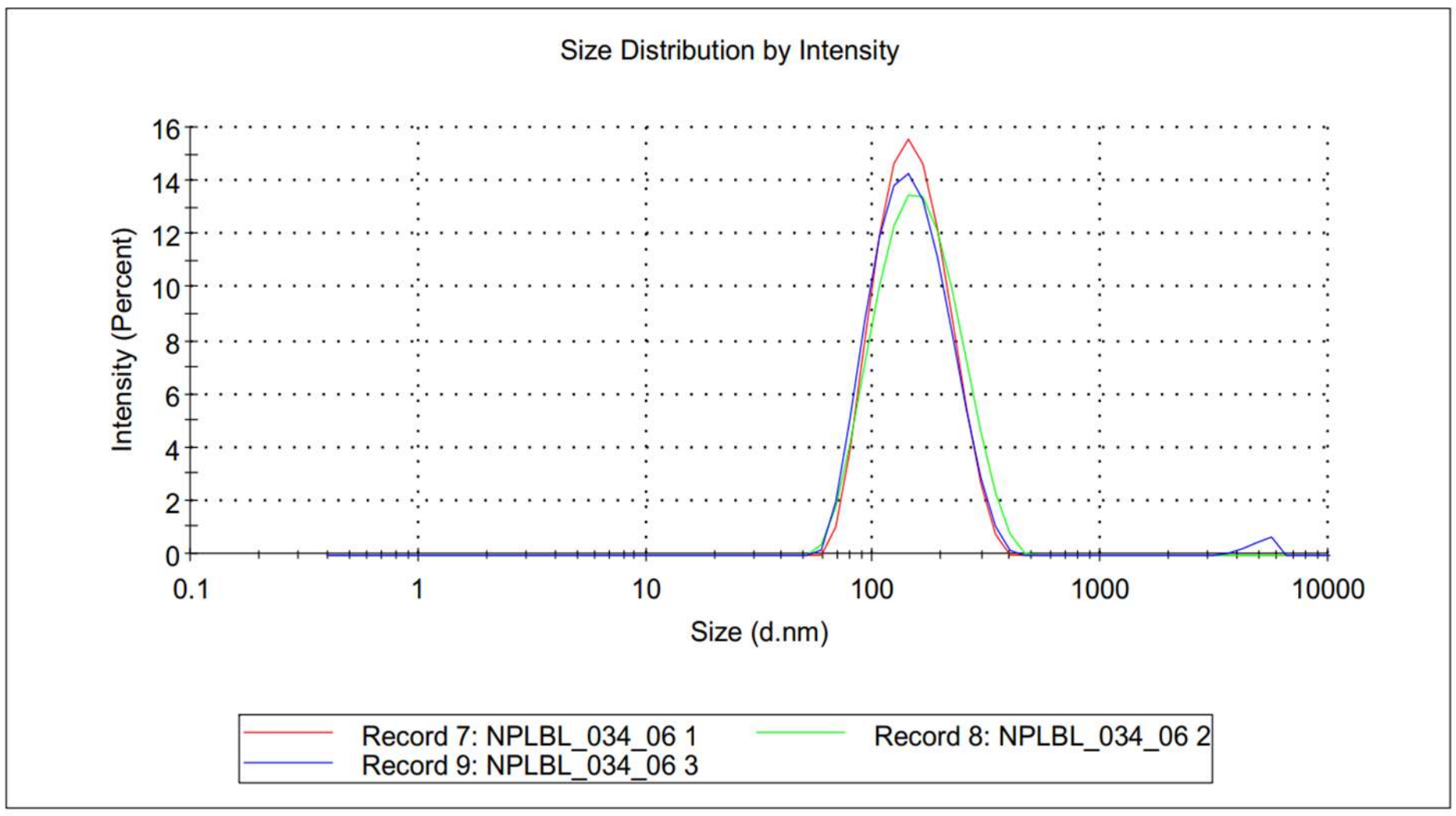
Size Distribution by Intensity
Intensity (Percent)
Size (d.nm)
Record 7: NPLBL_034_06 1
Record 8: NPLBL_034_06 2
Record 9: NPLBL_034_06 3

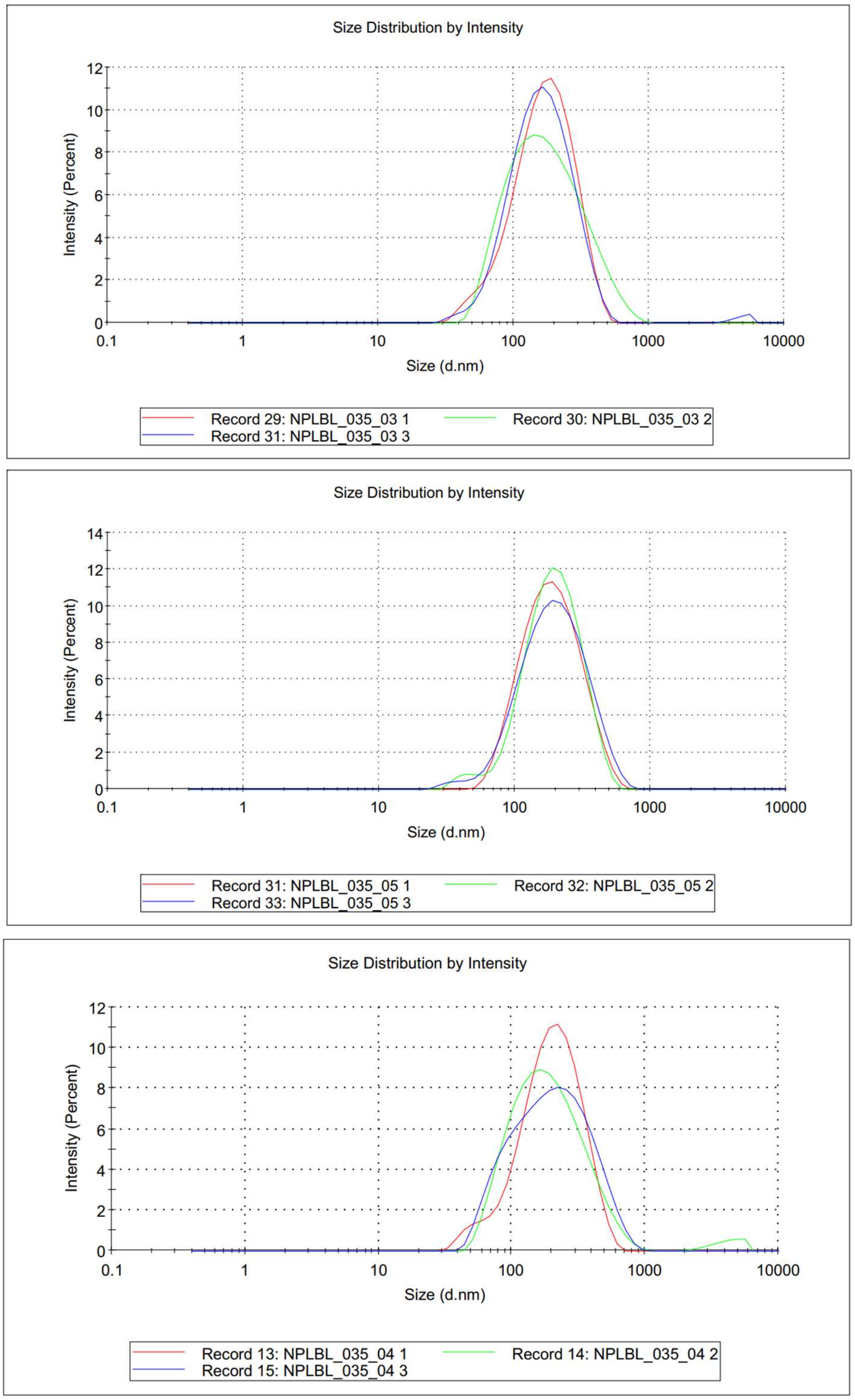

Dynamic Light Scattering Graphs (DLS) distributions of NLCs by intensity. Each graph respects a single batch with three technical replicates. Samples were diluted 1:200 in NaCl 10 mM. NPLBL refers to the blank NLCs, while NPLLV refers to levofloxacin-loaded NLCs. The numerical suffixes correspond to the specific surfactant used in the formulation: polysorbate 80 (NPLLV_033), poloxamer 407 (NPLLV_034), or poloxamer 188 (NPLLV_035).

Supporting Figure 2. Particle size distribution of NLCs measured by Nanotracking analysis (NTA).

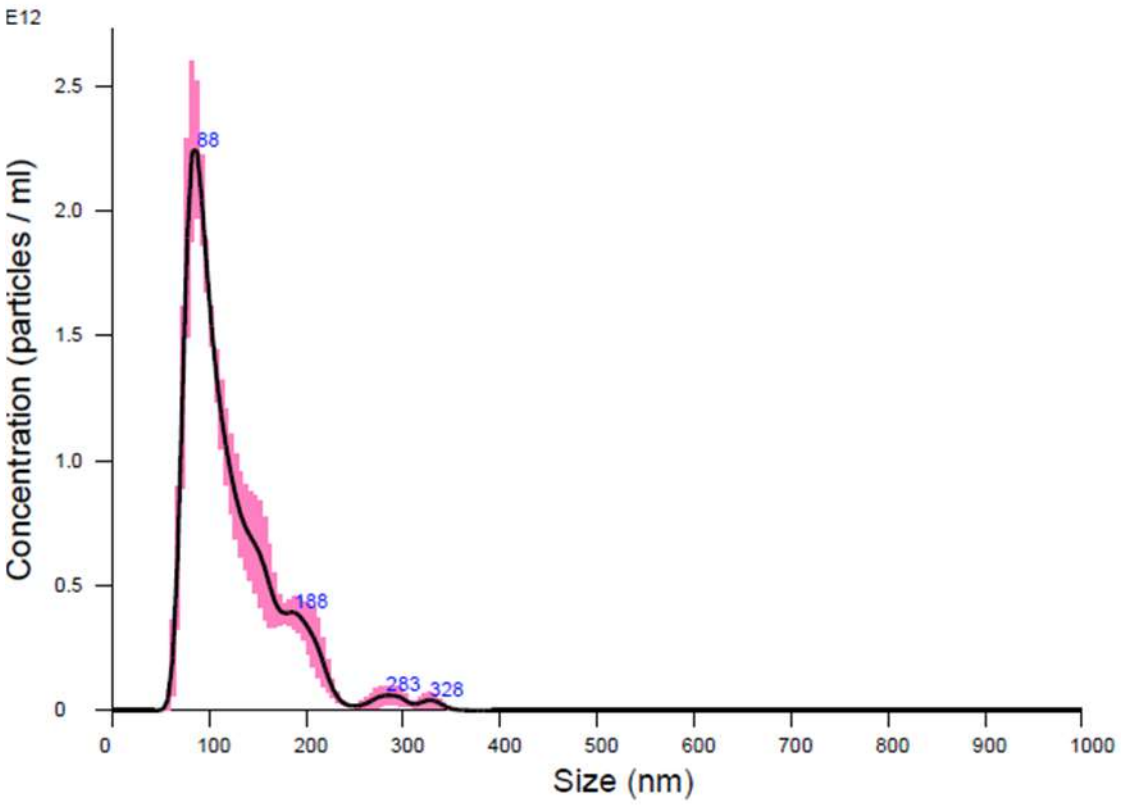

Averaged FTLA Concentration / Size for Experiment:
NPLBL_33_13 2013-09-09 18-08-01
Error bars indicate + / -1 standard error of the mean

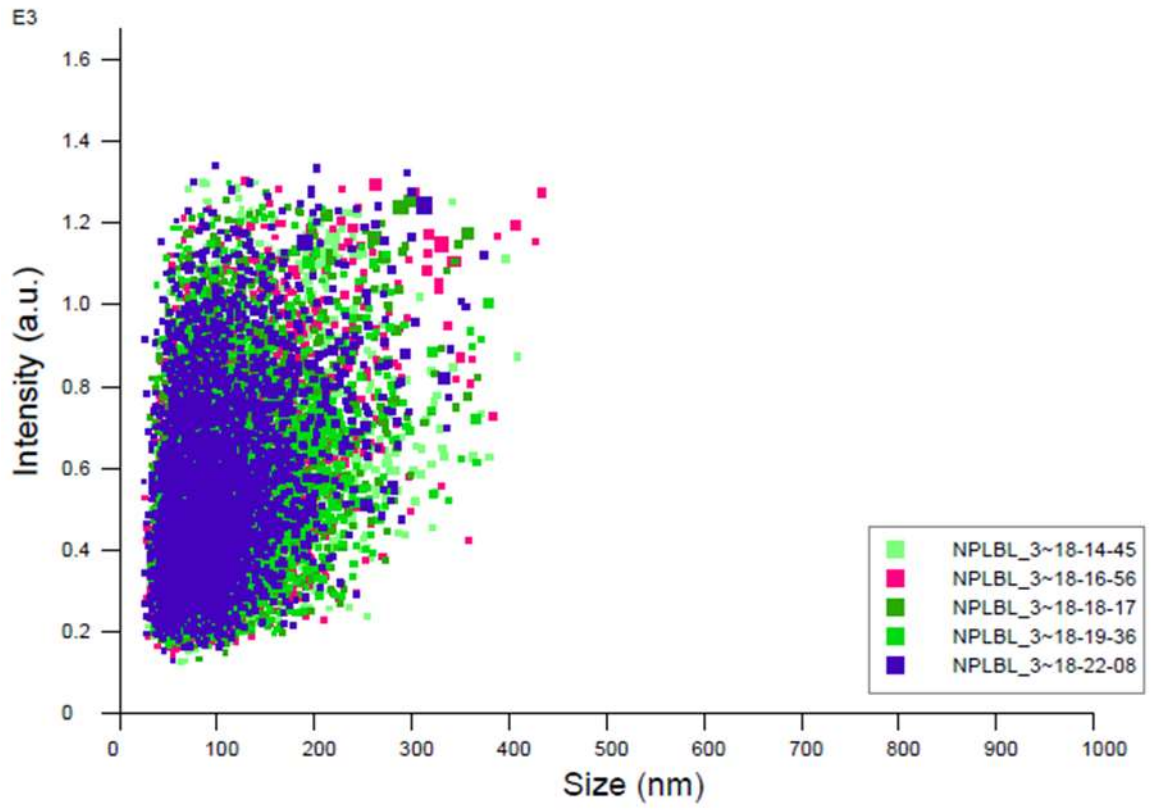

Intensity / Size graph for Experiment:
NPLBL_33_13 2013-09-09 18-08-01

A

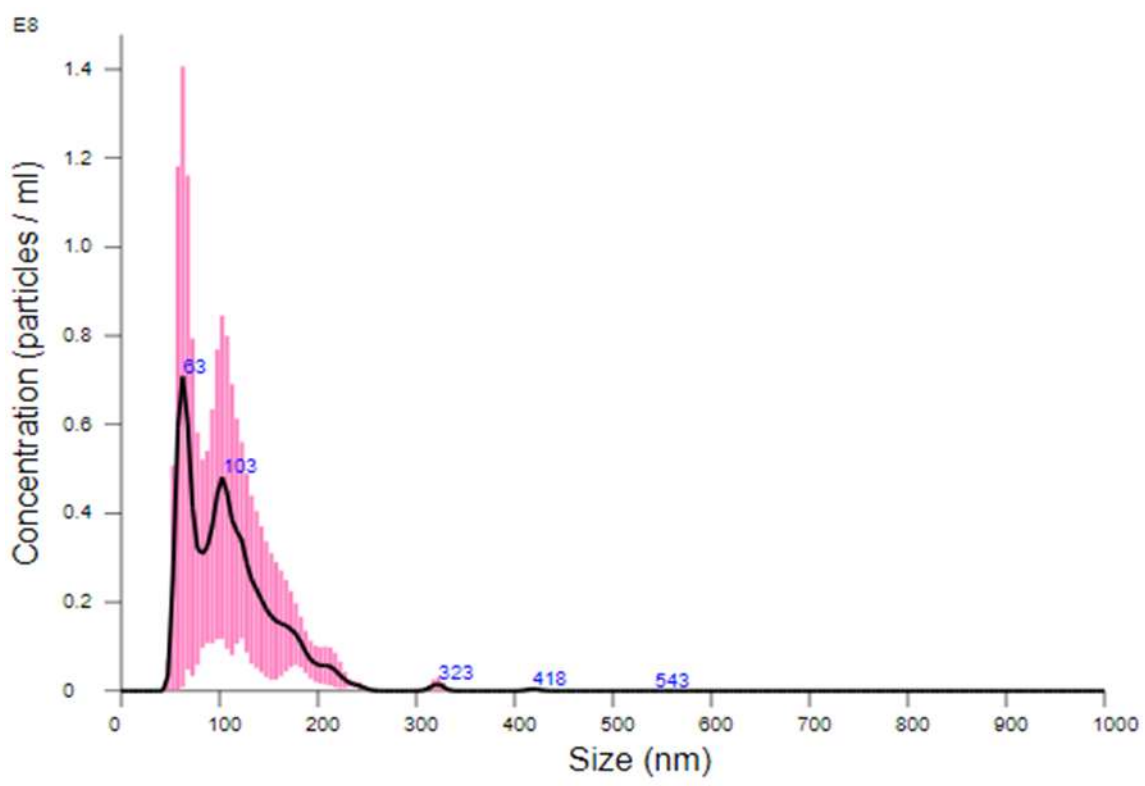

Averaged FTLA Concentration / Size for Experiment:
23023_03_16_NPLLV_033_014_Dil_15000 2013-09-09 18-58-26
Error bars indicate + / -1 standard error of the mean

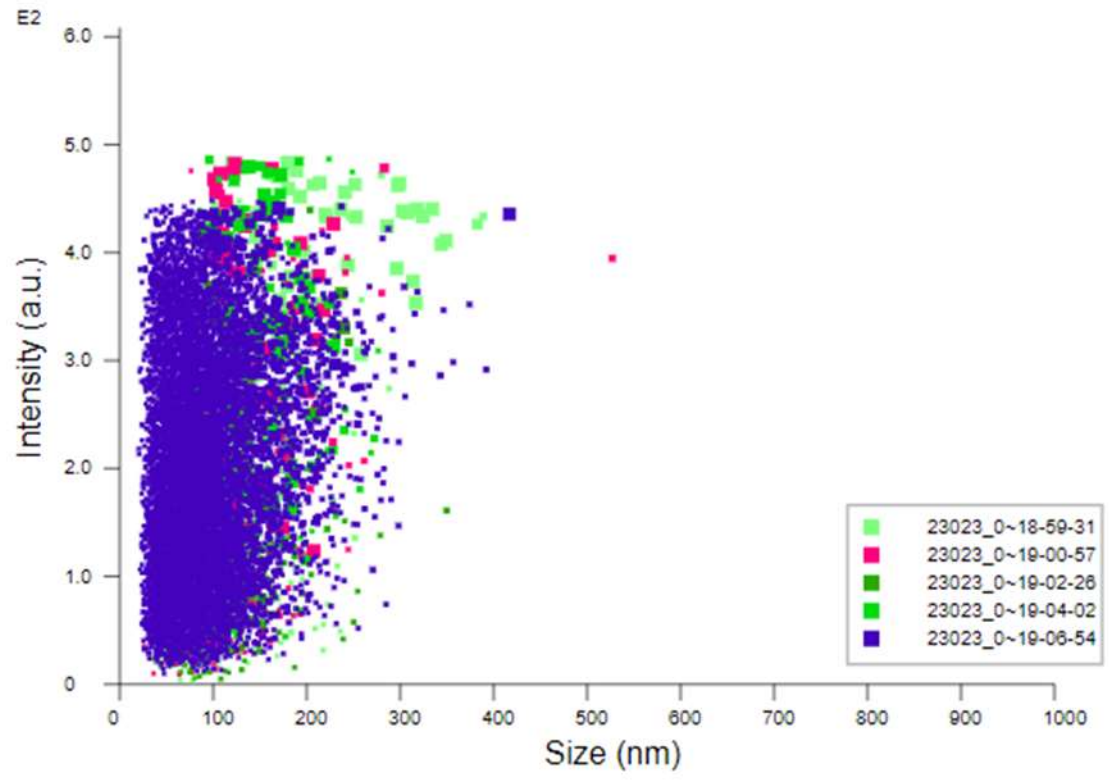

Intensity / Size graph for Experiment:
23023_03_16_NPLLV_033_014_Dil_15000 2013-09-09 18-58-26

B

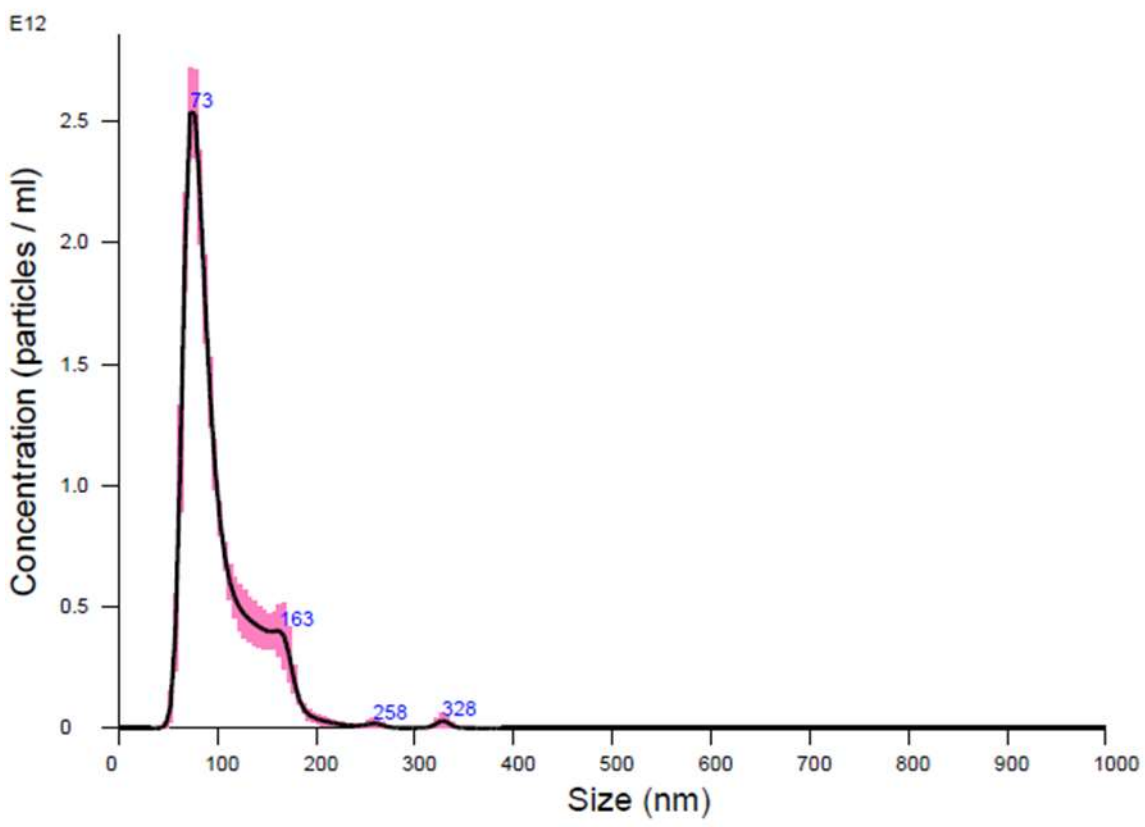

Averaged FTLA Concentration / Size for Experiment:
2023_03_16_NPLBL_034_05_Dil_15000 2013-09-09 19-24-05 20-13-09-09 20-26-42
Error bars indicate + / -1 standard error of the mean

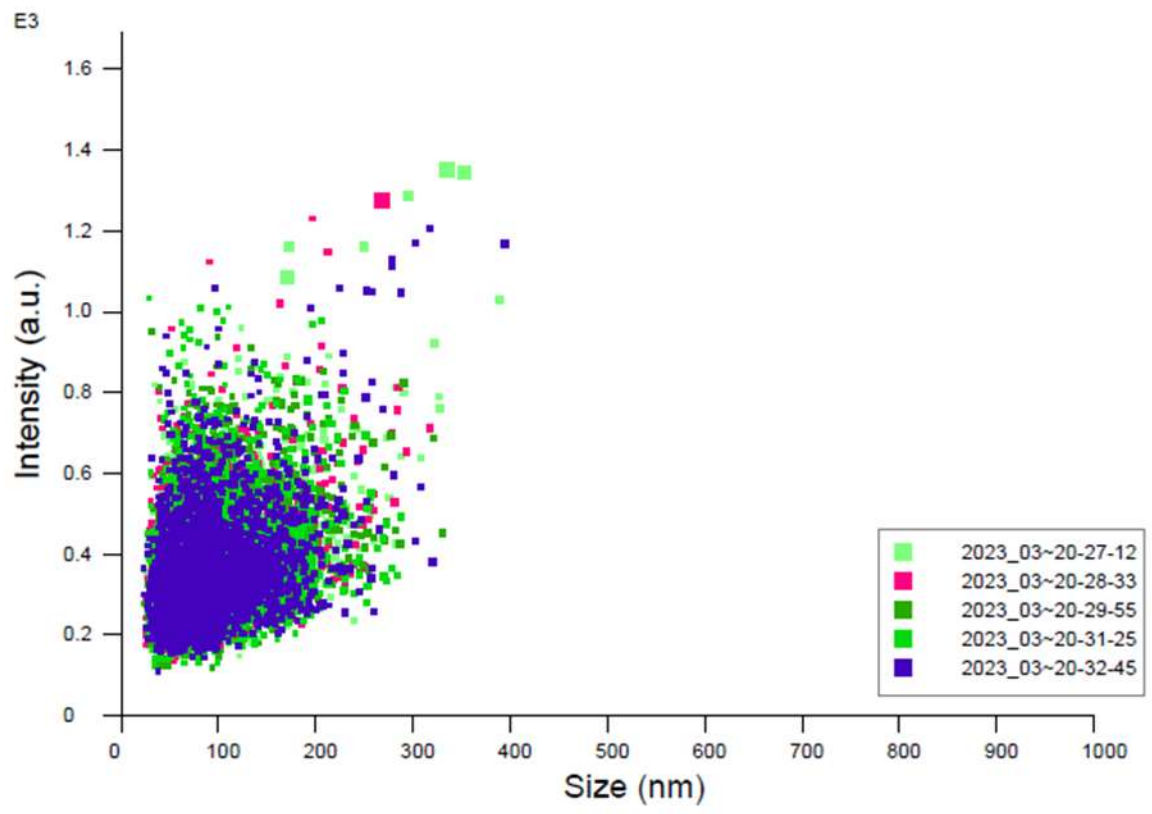

Intensity / Size graph for Experiment:
2023_03_16_NPLBL_034_05_Dil_15000 2013-09-09 19-24-05 20-13-09-09 20-26-42

C

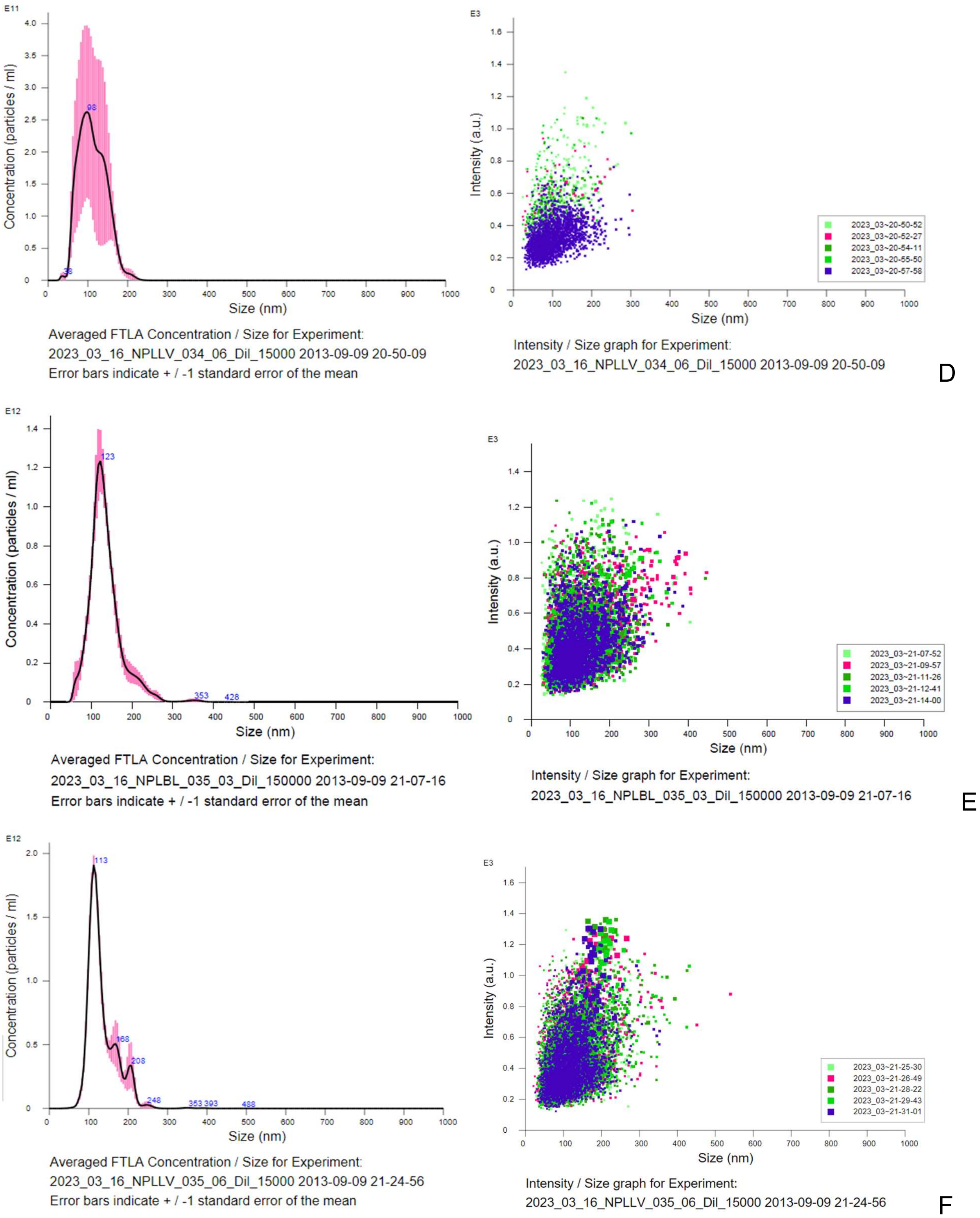

Concentration-size distributions (left) and particle size distributions by intensity (right) of NLCs formulated with polysorbate 80 without (A) and with (B) levofloxacin (LV) (NPLBL_033 and NPLLV_033, respectively); poloxamer 407 without (C) and with (D) levofloxacin (LV) (NPLBL_034 and NPLLV_034, respectively); and poloxamer 188 without (E) and with (F) levofloxacin (LV) (NPLBL_035 and NPLLV_035, respectively). Data were acquired via Nanoparticle Tracking Analysis (NTA). Prior to analysis, NLC samples were diluted 1:15,000 in ultrapure water.

]Supporting Table 1. Physicochemical characterization of NLCs' stability studies.

Nanoparticle	Condition	Z-Average (nm)	PDI	ZP (mV)	EE (%)	RI (%)
NPLLV_033 (P80)	**Fresh**	136.3±7.7	0.227±0.019	-19.5±2.3	77.1±3.1	2.2±1.6
	**40°C/75%UR**	303.7±70.4	0.323±0.018	-22.2±1.5	75.5±1.2	25.0±0.9
NPLLV_034 (P407)	**Fresh**	117.1±1.5	0.201±0.004	-11.5±0.7	88.6±2.0	0.8±0.9
	**40°C/75%UR**	114.1±6.8	0.189±0.023	-11.4±1.7	78.0±6.4	2.6±0.6
NPLLV_035 (P188)	**Fresh**	103.2±1.8	0.237±0.015	-16.0±0.1	82.5±5.2	2.5±2.1
	**40°C/75%UR**	102.2±3.0	0.233±0.020	-14.8±3.1	84.4±0.6	1.38±0.7

Nanoparticle stability was evaluated on the day of preparation and after 90 days of storage at 40 °C and 75% relative humidity (RH). The hydrodynamic diameter (Z-average), polydispersity index (PDI), and zeta potential (ZP) were determined by dynamic light scattering (DLS) in triplicate, following a 1:200 dilution in 10 mM NaCl. Encapsulation efficiency (EE) is relative to the amount of added drug, and related impurities (RI) levels are relative to the total chromatographic area, determined by high-performance liquid chromatography (HPLC), in duplicate or triplicate analyses.

Supporting Figure 3. Cell viability (Neutral red assay) of the non-differentiated Calu-3 cell line.

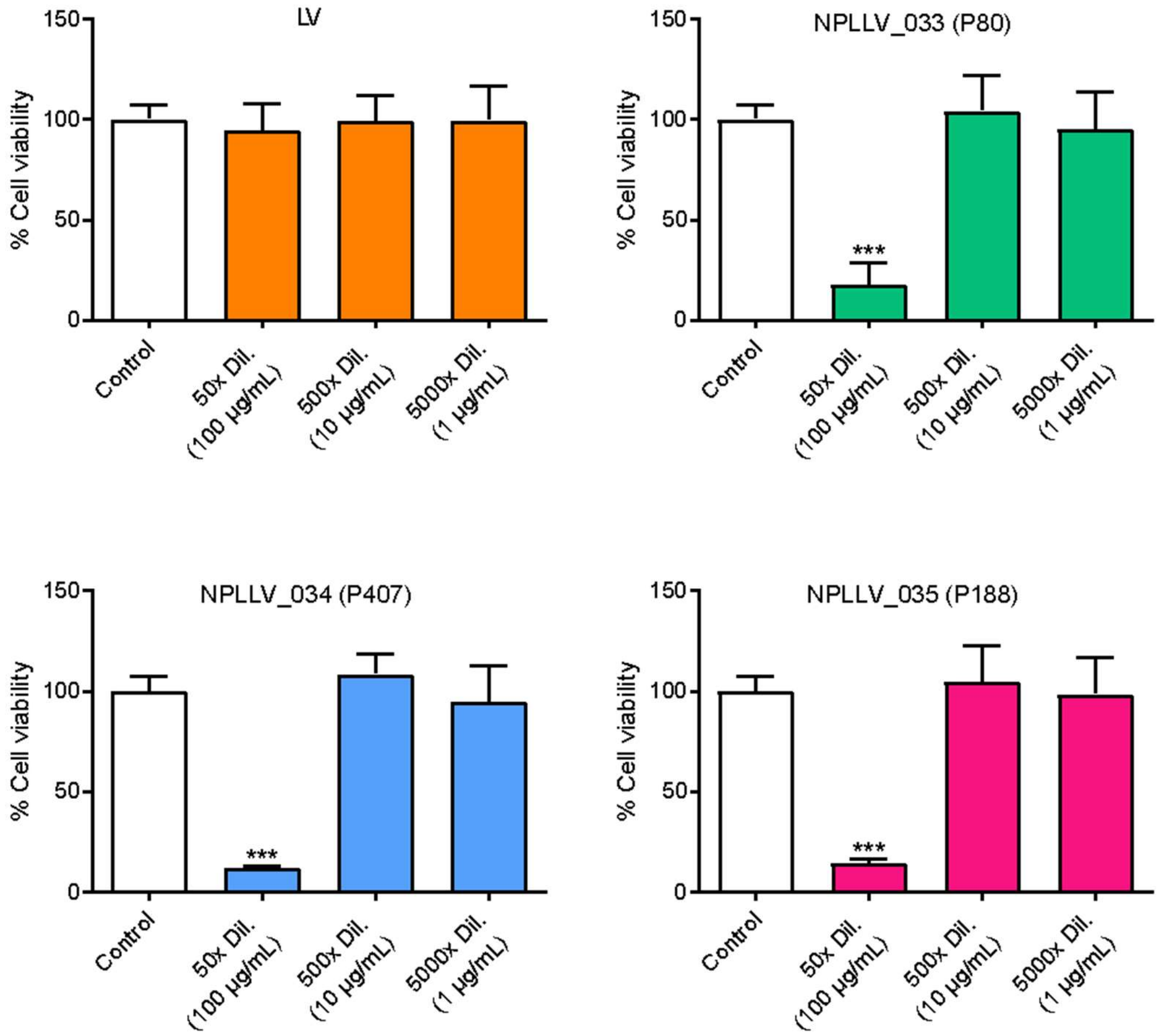

Calu-3 cell viability (%) following 24h of treatment. Cells were seeded in 96-well microplates (5 x 10^4 cells/well) and treated with polysorbate 80 (NPLLV_033; green bars), poloxamer 407 (NPLLV_034; blue bars), or poloxamer 188 (NPLLV_035; pink bars) alongside LV aqueous solution (orange bars) in three different concentrations (related to LV): 100, 10, or 1 µg/mL. All groups were compared with the positive control (DMEM medium, white bars). Graphs correspond to the percentage of Calu-3 cell viability after treatment with LV-loaded NLCs prepared Results are expressed as mean ± standard deviation, obtained from at least 2 independent assays, with n ≥ 3 replicates per assay. Symbol *** represents statistical significance compared to viable cell control ($p < 0.0005$), determined by the non-parametric Mann-Whitney test.

Supporting Figure 4. Characterization of differentiated Calu-3.

a. Methodology:

Post-differentiation Calu-3 were imaged using scanning electron microscopy. The cells were prepared based on the method previously described [1]. Calu-3 growth on transwells were fixed in 1.5% (v/v) glutaraldehyde in 0.1 M sodium cacodylate buffer for 1 h at 4 °C. Then, both apical and basal compartments were rinsed three times with sodium cacodylate buffer 0.1 M. The samples were then dehydrated with crescent concentrations of ethanol up to 100 % ethanol overnight. In the next day, membranes were cut from transwell inserts, dried at the critical point, mounted onto aluminium SEM stubs, and spattered with gold. Imaging was performed using a Quanta FEG 250 SEM microscope at 10 kV. The resulting micrographs reveal microvilli on the cell surface, indicating polarization and formation of these structures, similar to the alveolar environment, also seen in [1].

Fluorescence Microscopy was employed to confirm morphology and viability. Hoechst 33342 was used as a fluorescent stain for the DNA of fixed or living cells. Hoechst solution was prepared by diluting the stock solution in cell culture medium to a concentration of 2 µg/mL. A 100 µL volume of the diluted stain was added to the apical compartment of the transwell insert and incubated for 5 to 10 min, protected from the light. Fluorescence images were obtained with a Leica DM IL LED Inverted Laboratory Microscope equipped with 3-position fluorescence sliders (Leica Microsystems, Brazil) and qualitatively evaluated.

b. Images of differentiated Calu-3.

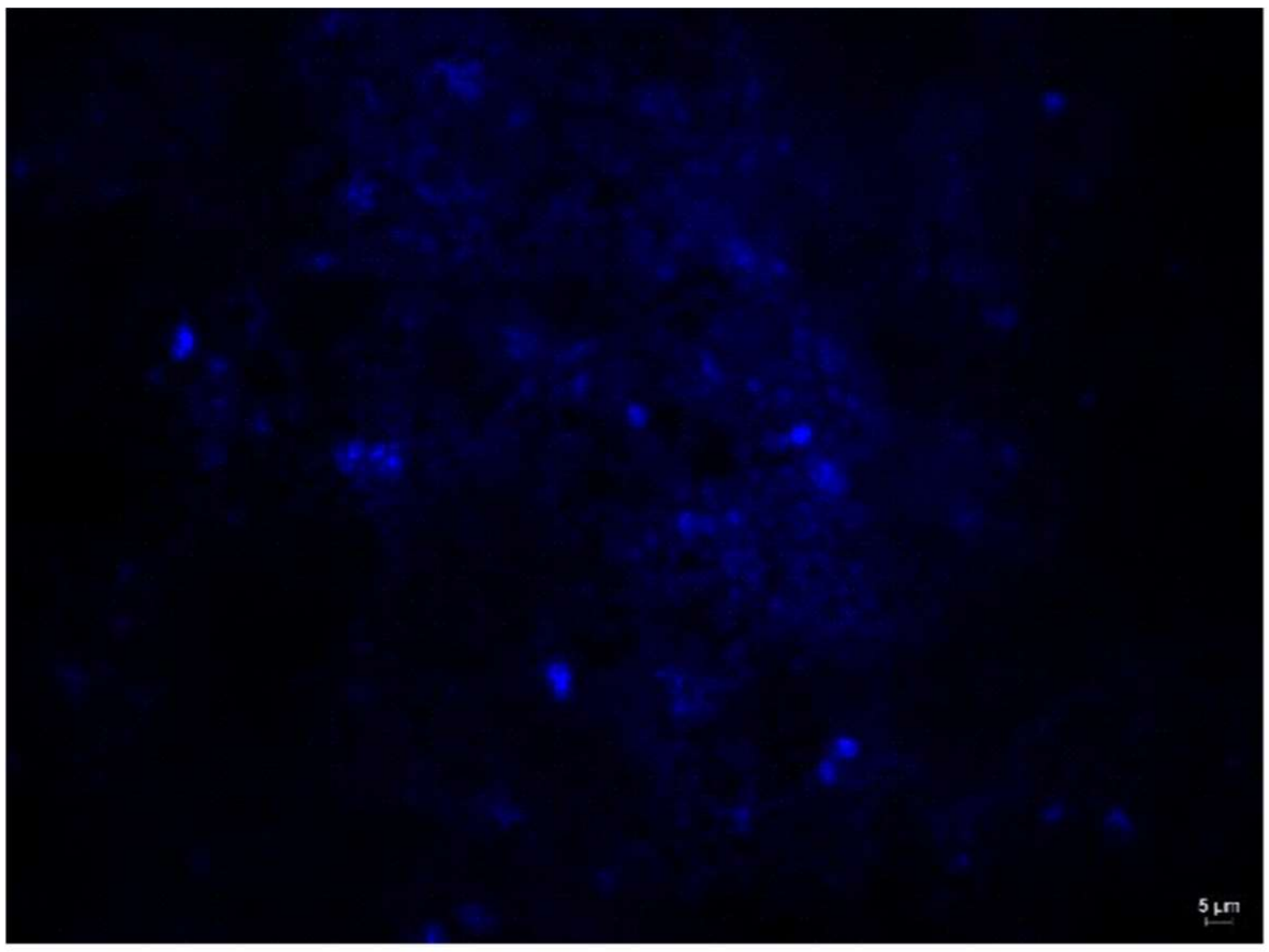

(a)

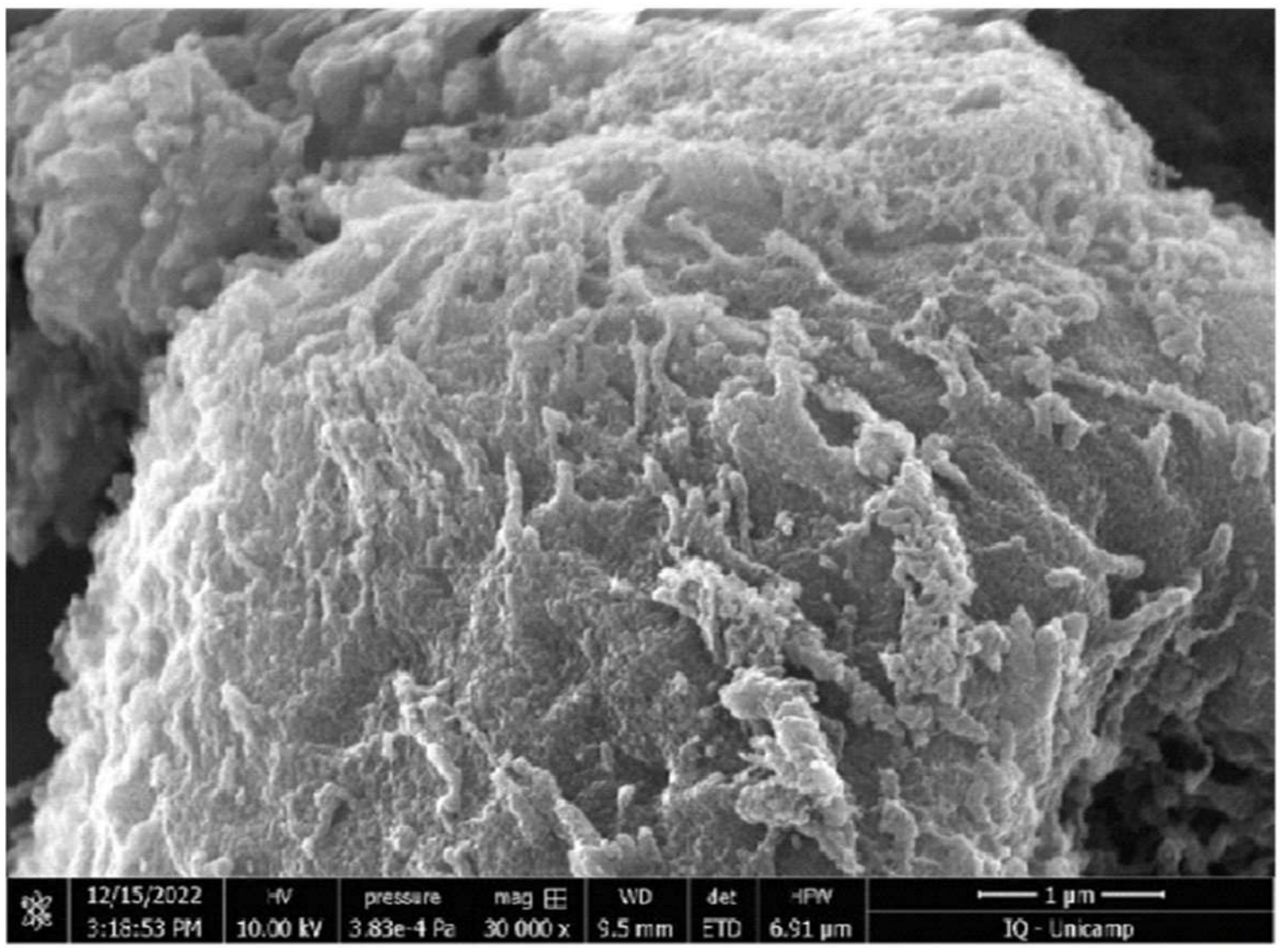

(b)

Morphological characterization of differentiated Calu-3 cell monolayer. (a) Fluorescence microscopy image of the Calu-3 monolayer with nuclei stained by Hoechst 33342. Images were obtained with a Leica DM IL LED Inverted Laboratory Microscope. Scale bar = 5 μm (a); Scanning electron microscopy (SEM) image of a differentiated Calu-3 epithelial layer grown at air-liquid interface. Cilia on the cell surface is visible, indicating cell differentiation (b). Scale bar = 1 μm. Magnification: 30,000x, WD = 9.5 mm, HV = 10.00 kV.

Supporting Figure 5. Particle size distribution of NPLLV_034 with p407, represented by number, before and after nebulization (A); nebulization setup (B).

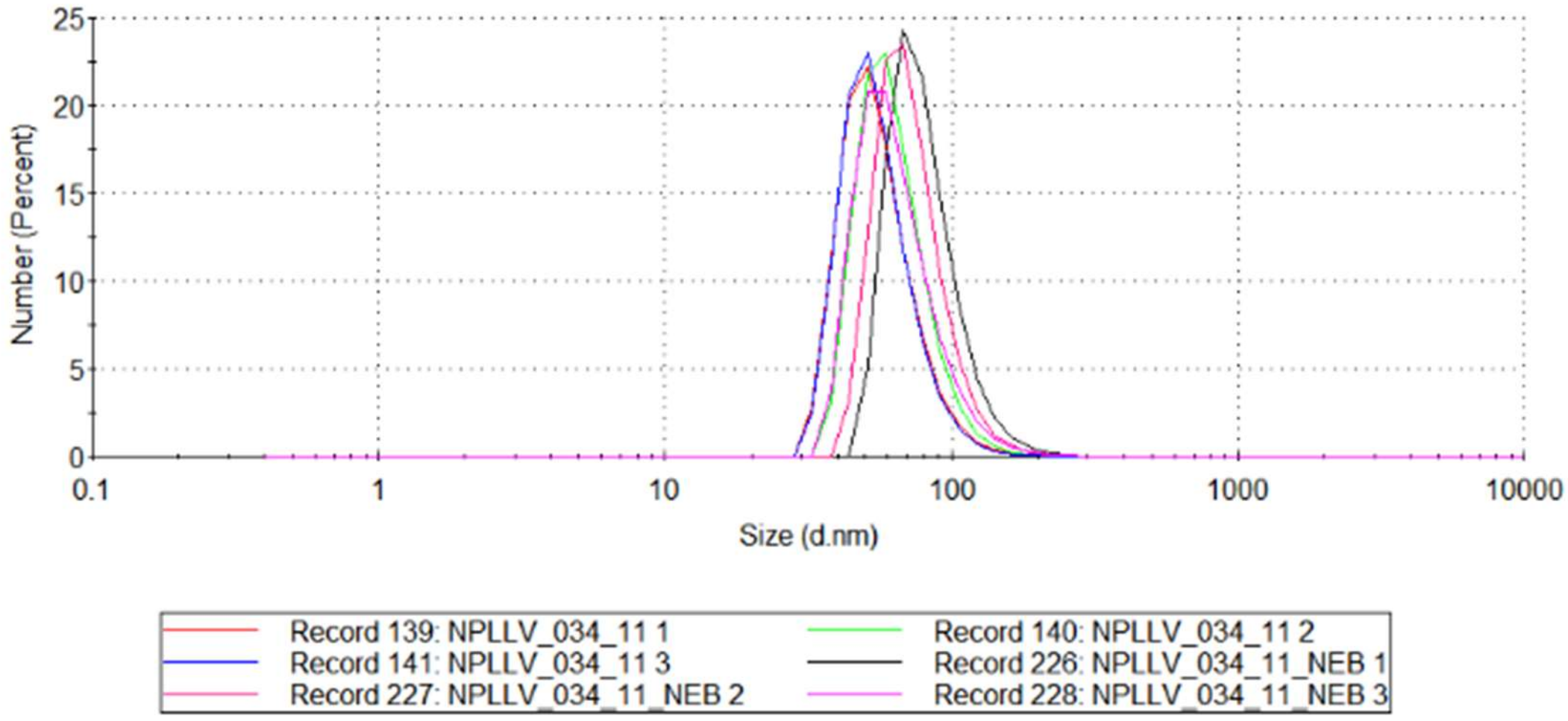

A

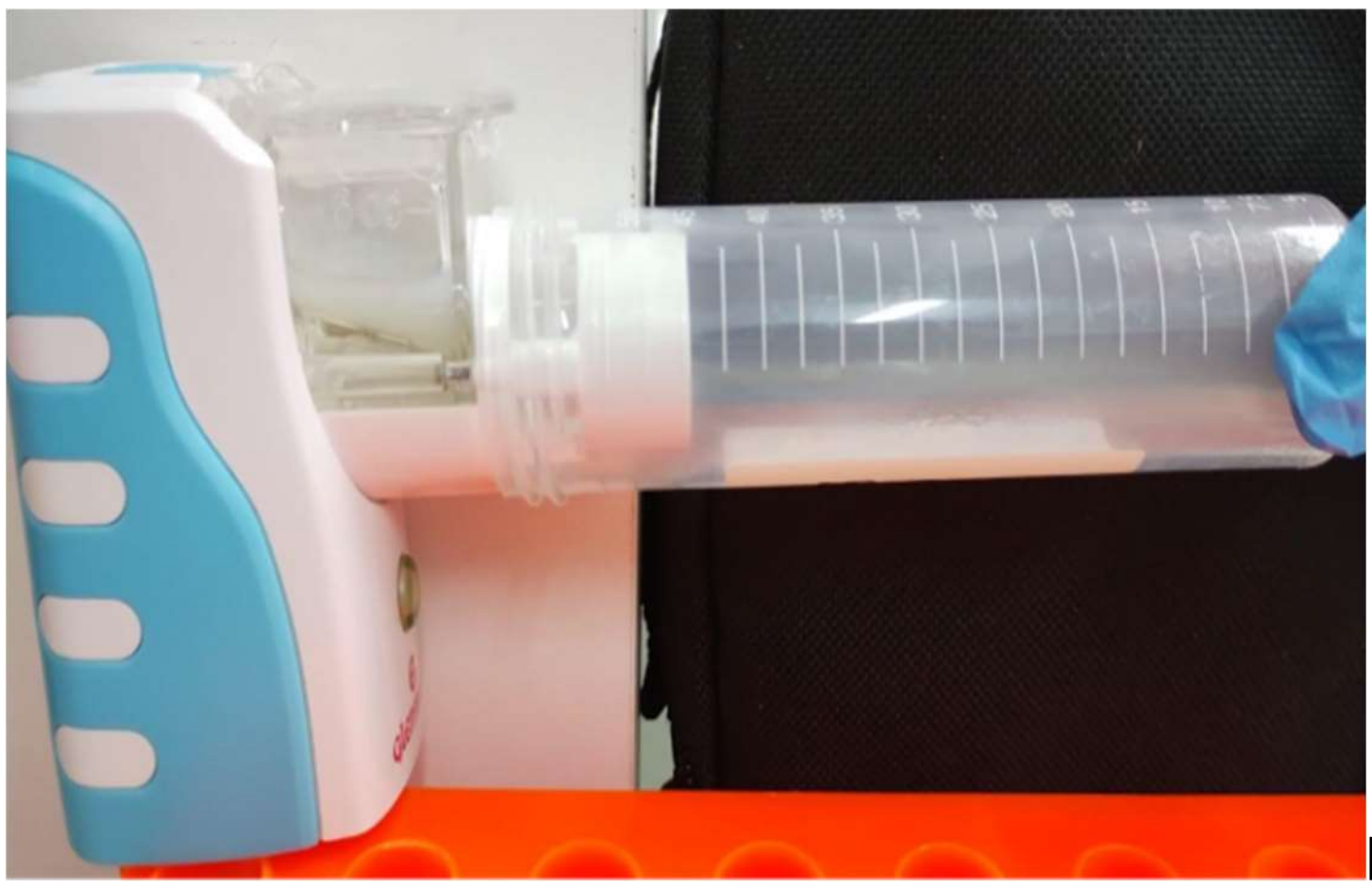

B

(A) Number-based particle size distribution of NPLLV_034 before and after nebulization. NLCs were diluted 1:200 in NaCl 10 mM prior to the analysis. (B) Experimental setup for nebulization using a mesh portable nebulizer (MBPN002, MicroBase Technology Corp., Taiwan). A volume of 3mL of the formulation was nebulized. The formulation (NPLLV_034) presented an adequate pH (5.84±0.33) for inhalation administration [2].

Supporting Table 2. Minimum Inhibitory Concentration (MIC) of placebo formulations and viability control

Bacterial strain	NPLBL_033 (µg/mL)	NPLBL_034 (µg/mL)	NPLBL_035 (µg/mL)	Viability Control (OD)
K. pneumoniae 700603	>6.25	>6.25	>6.25	0.654
K. pneumoniae BAA 1705	>1000	>1000	>1000	0.554
S. aureus ATCC 29213	>200	>200	>200	0.578
S. aureus ATCC 33591	>800	>800	>800	0.467
B. cepacia ATCC 25416	-	-	-	0.321
H. influenzae ATCC 51907	>10	>10	>10	1.269

Bacteria were treated with placebo formulations (blank NLCs) diluted to match the concentrations of the corresponding levofloxacin-containing formulations (n = 3 well replicates and at least two independent experiments). The placebos included NPLBL_033 with P80, NPLBL_034 with P407, and NPLBL_035 with P188. Placebos were not tested against *B. cepacia* ATCC 25416; however, based on results for the other bacteria, they are not expected to exhibit inhibitory activity. The viability control relates to wells of bacterial growth without inhibitors.

References:

(1) Cozens, D., Sutherland, E., Marchesi, F. *et al.* Temporal differentiation of bovine airway epithelial cells grown at an air-liquid interface. *Sci Rep* **8**, 14893 (2018). https://doi.org/10.1038/s41598-018-33180-w.

(2) Sahakijpijarn, S.; Smyth, H. D. C.; Miller, D. P.; Weers, J. G. Post-Inhalation Cough with Therapeutic Aerosols: Formulation Considerations. *Advanced Drug Delivery Reviews* **2020**, *165–166*, 127–141. https://doi.org/10.1016/j.addr.2020.05.003.
